# Insights into the intracellular localization, protein associations and artemisinin resistance properties of *Plasmodium falciparum* K13

**DOI:** 10.1371/journal.ppat.1008482

**Published:** 2020-04-20

**Authors:** Nina F. Gnädig, Barbara H. Stokes, Rachel L. Edwards, Gavreel F. Kalantarov, Kim C. Heimsch, Michal Kuderjavy, Audrey Crane, Marcus C. S. Lee, Judith Straimer, Katja Becker, Ilya N. Trakht, Audrey R. Odom John, Sachel Mok, David A. Fidock

**Affiliations:** 1 Department of Microbiology & Immunology, Columbia University Irving Medical Center, New York, NY, United States of America; 2 Department of Pediatrics, Washington University School of Medicine, St. Louis, MO, United States of America; 3 Department of Medicine, Columbia University Irving Medical Center, New York, NY, United States of America; 4 Biochemistry and Molecular Biology, Interdisciplinary Research Center, Justus Liebig University, Giessen, Germany; 5 Laboratory Imaging, Prague, Czech Republic; 6 Wellcome Sanger Institute, Wellcome Genome Campus, Hinxton, United Kingdom; 7 Department of Molecular Microbiology, Washington University School of Medicine, St. Louis, MO, United States of America; 8 Children’s Hospital of Philadelphia, Philadelphia, PA, United States of America; Francis Crick Institute, UNITED KINGDOM

## Abstract

The emergence of artemisinin (ART) resistance in *Plasmodium falciparum* intra-erythrocytic parasites has led to increasing treatment failure rates with first-line ART-based combination therapies in Southeast Asia. Decreased parasite susceptibility is caused by K13 mutations, which are associated clinically with delayed parasite clearance in patients and *in vitro* with an enhanced ability of ring-stage parasites to survive brief exposure to the active ART metabolite dihydroartemisinin. Herein, we describe a panel of K13-specific monoclonal antibodies and gene-edited parasite lines co-expressing epitope-tagged versions of K13 *in trans*. By applying an analytical quantitative imaging pipeline, we localize K13 to the parasite endoplasmic reticulum, Rab-positive vesicles, and sites adjacent to cytostomes. These latter structures form at the parasite plasma membrane and traffic hemoglobin to the digestive vacuole wherein artemisinin-activating heme moieties are released. We also provide evidence of K13 partially localizing near the parasite mitochondria upon treatment with dihydroartemisinin. Immunoprecipitation data generated with K13-specific monoclonal antibodies identify multiple putative K13-associated proteins, including endoplasmic reticulum-resident molecules, mitochondrial proteins, and Rab GTPases, in both K13 mutant and wild-type isogenic lines. We also find that mutant K13-mediated resistance is reversed upon co-expression of wild-type or mutant K13. These data help define the biological properties of K13 and its role in mediating *P*. *falciparum* resistance to ART treatment.

## Author summary

The development of drug resistance in *Plasmodium falciparum* parasites presents a significant impediment to the global fight against malaria. Partial resistance to artemisinin (ART), the core component of current first-line drugs, has swept across Southeast Asia. In *P*. *falciparum*-infected patients, ART-resistant parasites show slow rates of clearance following treatment with an ART derivative or ART-based combination therapy. Resistance to partner drugs has also emerged in Southeast Asia, leading to frequent treatment failures. Single amino acid mutations in the *P*. *falciparum* K13 protein constitute the primary genetic cause of ART resistance and predict an increased risk of treatment failure. By generating monoclonal antibodies, we have investigated the subcellular localization of K13 in dihydroartemisinin-treated or untreated parasites. Analytical microscopy data localize K13 to or near the endoplasmic reticulum and vesicles that mediate intracellular trafficking, including plasma membrane-associated cytostomes that import host hemoglobin into the parasite. Co-immunoprecipitation experiments with K13-specific monoclonal antibodies identified multiple proteins associated with the endoplasmic reticulum, vesicular trafficking, the cytosol, or the mitochondria, with no apparent differences between K13 mutant and wild-type parasites. We also observed that overexpression of mutant or wild-type K13 in K13 mutant parasites could restore susceptibility, supporting the hypothesis that K13 mutations cause loss of function.

## Introduction

Worldwide, malaria results in an estimated 400,000 or more fatalities each year, afflicting mostly infants and young children in sub-Saharan Africa [1]. Treatment of asexual blood-stage infections caused by *Plasmodium falciparum*, the most virulent human malaria parasite, relies on the efficacy of artemisinin (ART)-based combination therapies (ACTs). These first-line treatments pair a derivative of ART, an exceptionally fast-acting and potent antimalarial, with a longer-lived partner drug [2]. Commonly used partners include the arylaminoalcohol lumefantrine, used primarily in Africa, and piperaquine, a bisquinoline used predominantly in Southeast Asia [3].

In parasites, activation of ART or its derivatives requires iron-mediated reductive scission of the compound’s central endoperoxide bridge. The activator is thought to be primarily Fe^2+^ heme, a byproduct of parasite-mediated catabolism of host hemoglobin [4]. This cleavage event generates carbon-centered free radicals that can target proteins, lipids, nucleic acids, and heme itself, resulting in rapid cellular damage and parasite death [5–8]. ART is characterized by its ability to eliminate parasites from all stages of the intra-erythrocytic developmental cycle (IDC), including the young ring stages that form shortly after parasites invade host red blood cells (RBCs) [9,10]. This drug is highly potent against trophozoites that undergo maximal endocytosis and degradation of host hemoglobin, thus providing an abundant source of free heme.

Emerging resistance to ACTs threatens to reverse recent progress in reducing the global burden of malaria. Having first appeared in western Cambodia over a decade ago, resistance to ART is now nearly at fixation across Southeast Asia [11–15]. Clinically, ART resistance is defined as delayed parasite clearance after artesunate monotherapy or treatment with an ACT. As resistance to ART has become more widespread, selection pressure on partner drugs has increased. In some cases, delayed parasite clearance has escalated to treatment failure as partner drugs have succumbed to resistance [16]. In northeastern Thailand, a recent report documented 87% treatment failure rates with dihydroartemisinin (DHA) plus piperaquine [15].

*In vitro*, ART resistance is restricted to early ring-stage parasites, and is quantified as increased survival in the ring-stage survival assay (RSA_0-3h_), wherein parasites are exposed to a brief pulse (700 nM for 6h) of DHA. This assay distinguishes resistant parasites, which exhibit ≥1% survival after three days, from sensitive parasites that do not survive the pulse [17]. This resistance phenotype does not extend to the later trophozoite stage, presumably because parasites cannot counter the substantial toxicity arising from ART-mediated alkylation of the abundant heme moieties generated at this stage.

ART resistance is attributed primarily to individual point mutations in the parasite Kelch protein K13 [18]. Select mutations in K13 associate with delayed parasite clearance in *P*. *falciparum*-infected patients and with elevated survival in the RSA_0-3h_ [18,19]. These mutations all map to the protein’s carboxy-terminal six-bladed beta-propeller domain, a characteristic component of Kelch proteins that often serves as a scaffold for protein-protein interactions. Among the mutations examined *in vitro*, R539T and I543T confer the highest levels of ART resistance [20]. In contrast, the C580Y mutation confers only a modest degree of resistance, yet is the most prevalent in Southeast Asia [20,21]. This finding has been attributed to the relatively minimal fitness cost conferred by the C580Y mutation in Southeast Asian parasites [22].

In addition to its propeller domain, K13 comprises an apicomplexan-specific domain of unknown function and a BTB/POZ dimerization domain. The latter is found in a subset of Kelch proteins that commonly mediate ubiquitin-dependent protein degradation via the proteasome by serving as substrate adaptors for E3 ubiquitin ligases [18,23]. K13 shows homology with the mammalian BTB-Kelch protein Keap1, which controls the cell’s adaptive response to oxidative stress [18,24]. In *P*. *falciparum*, the *K13* gene appears to be essential based on conditional knockout experiments showing that K13-deficient parasites do not progress past the ring stage and transition into non-viable condensed forms, and by a large-scale saturation mutagenesis study that observed no disruptions in the *K13* coding region [25,26].

Mechanistic studies have led to several proposals for how mutant K13 might counter ART-mediated cellular toxicity. These proposals include lowering the levels of the heme activator of ART including via reduced hemoglobin endocytosis in rings [27–30], upregulating endoplasmic reticulum (ER) stress-response pathways [31], reducing the levels of ubiquitinated proteins [32], promoting translational arrest via differential phosphorylation of the translation initiation factor eIF2α [33], or increasing levels of the phospholipid phosphatidylinositol‐3‐phosphate (PI3P) [34,35]. To gain additional insight into the biology of this protein, we raised K13-specific monoclonal antibodies (mAbs) and used these to interrogate this protein’s subcellular localization in DHA-exposed or vehicle-treated asexual blood-stage parasites. Using co-immunoprecipitation (co-IP), we also identified potential interactions with other parasite proteins and examined their predicted roles in *P*. *falciparum* metabolism and development. Results presented herein implicate K13 in multiple cellular functions, including vesicular trafficking and ER homeostasis, and suggest an unexpected association with the mitochondria upon DHA treatment.

## Results

### K13 localizes to the endoplasmic reticulum and to vesicular structures

**Fig 1:**
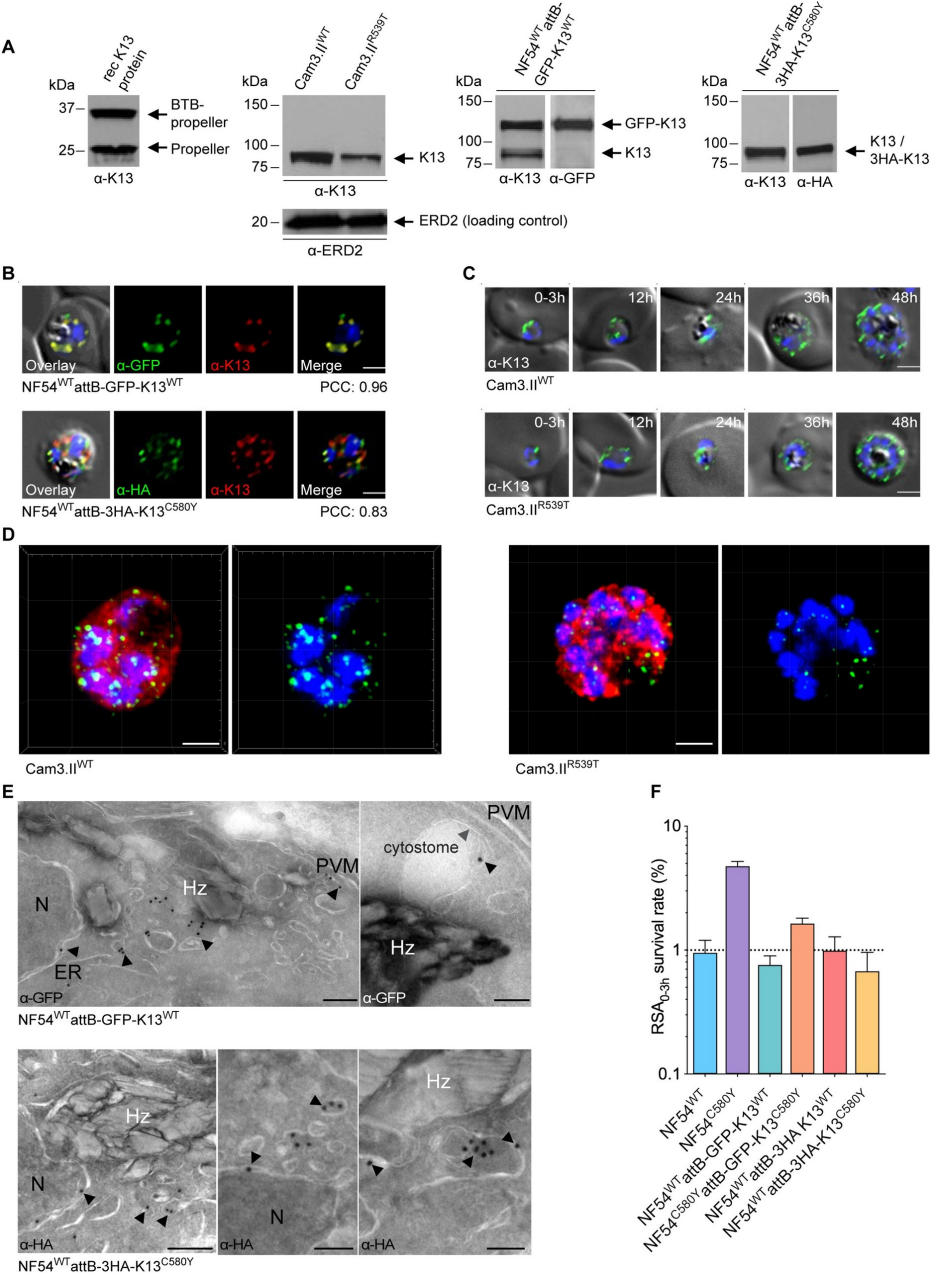
The *P*. *falciparum* artemisinin resistance determinant K13 localizes to the parasite ER and intracellular vesicles. **(A)** Western blots probed with the E9 monoclonal antibody (mAb) raised against the K13 propeller domain. Left to right: recombinant K13 protein fragments used as immunogens; Cam3.II^WT^ and Cam3.II^R539T^ asexual blood-stage parasite extracts; and NF54^WT^attB-GFP-K13^WT^ and NF54^WT^attB-3HA-K13^C580Y^ extracts. ERD2 was used as a loading control for the Cam3.II lines. The NF54^WT^attB-GFP-K13^WT^ and NF54^WT^attB-3HA-K13^C580Y^ blots were also probed with anti-GFP or anti-HA antibodies, respectively. **(B)** Immunofluorescence assay (IFA) images showing K13 localization in NF54^WT^attB-GFP-K13^WT^ (top) and NF54^WT^attB-3HA-K13^C580Y^ (bottom) trophozoites. Parasites were co-stained with the K13 E3 mAb and antibodies specific to GFP or HA. Pearson correlation coefficient (PCC) values indicate the degree of spatial co-localization between the two signals and were calculated by determining the fluorescence intensity correlations of Alexa Fluor 488 (anti-GFP or anti-HA) and 594 (K13 mAb). Nuclei were stained with DAPI (blue). Scale bars: 2 μm. **(C)** IFA images depicting K13 localization in Cam3.II^WT^ (top) and Cam3.II^R539T^ (bottom) parasites throughout asexual blood-stage development. Parasites were stained with the K13 E3 mAb. Sampling was performed every 12h, beginning with tightly synchronized 0–3 hpi ring-stage parasites. Scale bars: 2 μm. (**D**) Super resolution microscopy of mature parasites labeled with antibodies to K13 (green), the cytosolic marker HAD1 (red) and the nuclear stain DAPI (blue), showing K13-positive punctate foci. Scale bars: 2 μm. A video representation is shown in **S1 Video**. **(E)** Representative immunoelectron microscopy (IEM) images depicting K13 localization in NF54^WT^attB-GFP-K13^WT^ or NF54^WT^attB-3HA-K13^C580Y^ parasites stained with 18 nm colloidal gold-conjugated anti-GFP or anti-HA antibodies. Arrowheads highlight locations of interest. ER, endoplasmic reticulum; Hz, hemozoin; N, nucleus; PVM, parasitophorous vacuolar membrane. Scale bars: 100 nm. **(F)** Ring-stage survival assay (RSA_0-3h_) results from NF54^WT^attB-GFP-K13^WT^, NF54^C580Y^attB-GFP-K13^C580Y^, NF54^WT^attB-3HA-K13^WT^ and NF54^WT^attB-3HA-K13^C580Y^ transgenic lines, compared to the sensitive and resistant benchmarks NF54^WT^ and NF54^C580Y^, respectively. Data show mean ± SEM percent survival of 700 nM dihydroartemisinin (DHA)-treated early ring-stage parasites (0–3 hpi) compared with control dimethyl sulfoxide (DMSO)-treated parasites processed in parallel. Experiments were performed on 2–6 independent occasions with technical duplicates.

To probe the subcellular localization of K13, we raised monoclonal antibodies (mAbs) by immunizing mice with recombinant protein fragments consisting of either the K13 propeller domain alone or the propeller domain plus the upstream BTB/POZ domain, and cloning K13-specific hybridoma populations. Western blot screening identified the E9 clone that recognized the two recombinant K13 immunogens (bands at ~25 kDa and ~35kDa for the propeller domain alone or propeller plus BTB/POZ domains, respectively) (**Fig 1A**). In parallel, we generated recombinant NF54^WT^attB parasite lines that express endogenous wild-type (WT) K13 and co-express stably-integrated transgenic copies of WT or C580Y K13, which were N-terminally tagged with GFP or 3HA, respectively. These lines are referred to herein as NF54^WT^attB-GFP-K13^WT^ or NF54^WT^attB-3HA-K13^C580Y^ (**Table 1** and **S1A–S1C Fig**).

**Table 1:** *Plasmodium falciparum* lines employed in this study.

Name	Strain	Endogenous *K13* locus	Transgene	Transgene 5' UTR
NF54^WT^	NF54	WT	--	--
NF54^C580Y^	NF54	C580Y	--	--
NF54^WT^attB-GFP-K13^WT^	NF54attB	WT	GFP-K13^WT^ (integrated into *cg6* attB)	*K13*
NF54^C580Y^attB-GFP-K13^C580Y^	NF54attB	C580Y	GFP-K13^C580Y^ (integrated into *cg6* attB)	*K13*
NF54^WT^attB-3HA-K13^WT^	NF54attB	WT	3HA-K13^WT^ (integrated into *cg6* attB)	*pbef1α*
NF54^WT^attB-3HA-K13^C580Y^	NF54attB	WT	3HA-K13^C580Y^ (integrated into *cg6* attB)	*pbef1α*
Cam3.II^R539T^	Cam3.II	R539T	--	--
Cam3.II^WT^	Cam3.II	WT	--	--
Cam3.II^C580Y^	Cam3.II	C580Y	--	
CamWT	CamWT	WT	--	--
CamWT^C580Y^	CamWT	C580Y	--	--
Dd2^WT^ GFP-Rab6	Dd2	WT	Rab6-GFP (episome)	*pfsec12*
Dd2^R539T^ GFP-Rab6	Dd2	R539T	Rab6-GFP (episome)	*pfsec12*
Dd2^WT^ Sec24A-GFP	Dd2	WT	Sec24A-GFP (episome)	*pfsyntaxin17*
3D7^WT^ Mito-hGrx1-roGFP2	3D7	WT	Mito-hGrx1-roGFP2	*pfcrt*

We tested our E9 mAb by Western blot against asynchronous parasite extracts from the contemporary Cambodian isolate Cam3.II that carries the K13 R539T mutation (referred to herein as Cam3.II^R539T^) and its isogenic, gene-edited K13 WT counterpart Cam3.II^WT^ [20]. This K13 mAb clearly labeled WT and mutant K13 (both at ~85 kDa), with evidence of reduced K13 labeling in Cam3.II^R539T^ parasites (**Fig 1A** and **S1D Fig**). Quantification provided evidence of a slight reduction in K13 protein levels in the Cam3.II^C580Y^ and Cam3.II^R539T^ synchronized ring-stage parasites (these decreases were estimated at ~24% and ~34%, respectively) compared to WT levels (**S1E Fig**). We also tested the GFP- or HA-tagged NF54^WT^attB lines expressing K13 *in trans*, revealing bands at ~110 kDa for GFP-K13^WT^ and ~86 kDa for 3HA-K13^C580Y^ (**Fig 1A** and **S1D Fig**) that were also recognized by anti-GFP- or anti-HA antibodies respectively.

Our K13-specific E9 mAb was found to be suitable only for Western blots and to not provide a robust signal by immunofluorescence assay (IFA). Further screening led us to identify a second K13-specific mAb, clone E3, which was suitable for IFAs (but not Western blots). This mAb was tested against the parasite lines NF54^WT^attB-GFP-K13^WT^ and NF54^WT^attB-3HA-K13^C580Y^. These assays allowed us to calculate the overlap coefficient (termed the Pearson correlation coefficient, PCC) between the signal from our K13 E3 mAb and that from anti-GFP or anti-HA antibodies tested against epitope-tagged K13 proteins. PCC values range from +1 (indicating complete overlap) to 0 (random association) to -1 (mutually exclusive signals with zero overlap). In both the GFP and HA tagged lines we observed a very high degree of correlation between the signals from the anti-K13 mAb E3 and anti-GFP or anti-HA antibodies, with PCC values of 0.96 and 0.83, respectively (**Fig 1B**). These Western blot and IFA results with native and epitope or fluorescent protein-tagged lines validated the specificity of our K13-specific mAbs.

Using our E3 mAb, we next examined the subcellular localization of K13 throughout the IDC by IFA. These assays were performed on tightly synchronized parasites and used the Cam3.II^WT^ and Cam3.II^R539T^ isogenic lines. Samples were collected every 12h beginning with early rings (0-3h post invasion (hpi)). Early rings showed a single K13-positive focus within the parasite cytosol in both lines. As parasites progressed into schizonts, the number of K13-positive foci increased. Whereas the majority of these foci appeared to be evenly distributed throughout the parasite cytosol, others appeared to be proximal to specific organelles including the parasite plasma membrane, the ER, and the digestive vacuole (DV). No differences in K13 localization were evident between WT and mutant parasites (**Fig 1C**). Super-resolution microscopy clearly showed multiple K13 foci in trophozoites of both K13 mutant and WT parasites (**Fig 1D; S2A and S2B Fig**). Three-dimensional rotations suggested elongated, tunnel-like shapes that might link subcellular compartments (**S1 Video**). Quantification of the number of visualized K13 foci estimated a 48% reduction in Cam3.II^R539T^ trophozoites compared with Cam3.II^WT^ trophozoites (**S2C Fig**).

We also examined K13 localization using immunoelectron microscopy (IEM; **Fig 1D**). These studies were conducted with NF54^WT^attB-GFP-K13^WT^ and NF54^WT^attB-3HA-K13^C580Y^ parasites, with K13 detected via anti-GFP or anti-HA colloidal gold-conjugated primary antibodies. In trophozoites, K13 appeared to localize to the parasite cytosol, often within small vesicles, vesicular clusters, or tubulovesicular networks that likely belong to the ER or the Golgi apparatus. K13 was also frequently associated with the ER itself, as well as with the plasma and nuclear membranes and the DV. We also saw evidence of K13 associating with cytostomes that traffic host-endocytosed hemoglobin from the parasitophorous vacuolar space to the DV (**Fig 1E**).

### K13 overexpression restores artemisinin sensitivity to K13-mutant parasites

Our epitope-tagged lines made it possible to further explore the relationship between co-expression of mutant or WT K13 *in trans* and endogenous mutated or wild-type K13. The impact of co-expression on *in vitro* resistance was measured using the RSA_0-3h._ As sensitive and resistant benchmarks, NF54^WT^ and NF54^C580Y^ parasites (expressing K13 WT or C580Y respectively) yielded mean RSA survival values of 1.0% and 4.8% respectively (**Fig 1F**). Co-expression of GFP-K13^WT^ or 3HA-K13^C580Y^ in our NF54^WT^attB epitope-tagged lines (see above) led to ART sensitivity, with RSA values of 0.8% and 0.7% in the NF54^WT^attB-GFP-K13^WT^ and NF54^WT^attB-3HA-K13^C580Y^ lines, respectively. As comparators, we also engineered an NF54^C580Y^attB line co-expressing GFP-K13^C580Y^ as well as an NF54^WT^attB line co-expressing 3HA-K13^WT^ (**Table 1**). The former line expresses mutant K13 in both the endogenous and transgene loci and demonstrated a nominally less sensitive phenotype (with 1.6% mean RSA survival), relative to the fully sensitive NF54^WT^ line. The latter line expresses WT K13 in both loci and was fully sensitive (mean RSA survival 1.0%). These data confirm that mutant K13 does not confer resistance in a dominant-negative manner and suggest that overexpression of the mutant protein mostly reverts K13 mutant parasites to DHA sensitivity. Our results agree with recently published evidence that the RSA_0-3h_ phenotype inversely correlates with K13 abundance and that K13 levels are reduced in mutant parasites relative to WT [27,28].

### K13 co-immunoprecipitates with vesicular transport, ER, and mitochondrial proteins

To identify putative K13-interacting partners, we performed six independent HPLC/MS-MS-based co-IP experiments using two K13 mAbs (E3 and D9, which both yielded robust IFA signals). These experiments comprised 13 test samples from parasite cultures enriched in 0–12 hpi rings. These samples were prepared from Cam3.II lines expressing WT, C580Y or R539T K13, as well as from CamWT lines expressing WT or C580Y K13 (**Table 1** and **S1 Table**). For a given sample, we retained only proteins that were identified by ≥3 peptide spectra. We then filtered results across all samples by retaining only proteins that were present in ≥3 of the 6 independent experiments and ≥5 of the 13 test samples, and absent in all of the 10 negative control samples (i.e. that used an unrelated antibody, or used affinity columns without the addition of anti-K13 antibodies). Results showed that K13 was by far the most abundant protein detected, representing 22% of the total number of spectra detected in our filtered list of 83 high-confidence immunoprecipitated proteins (**Table 2**). In a secondary analysis, we relaxed the criteria to allow for proteins that appeared in 1 to 3 negative control samples (out of 10), while retaining the positive criteria listed above. This yielded an additional 90 proteins as putative interactors (**S2 Table**).

**Table 2:** Putative K13-interacting protein partners identified by co-immunoprecipitation and LC/MS-MS.

PlasmoDB Gene ID	Gene Name	Abbreviation	Cellular component and/or functional features	Mean % total spectral counts, normalized	Number of experiments present (of 6)^1^	Number of samples present (of 13 total, 6 WT, 7 mutant)^2^
PF3D7_1343700	Kelch protein K13	K13	Putative CUL3 ubiquitin ligase adaptor protein	22.19%	6	13 (6,7)
PF3D7_0922200	S-adenosylmethionine synthetase	SAMS	Methionine metabolism	5.19%	3	6 (3,3)
PF3D7_1120100	Phosphoglycerate mutase 1, putative	PGM1	Glycolysis	3.58%	4	8 (4,4)
PF3D7_0826700	Receptor for activated c kinase	RACK	Cytosolic multi-functional scaffolding protein	2.47%	5	9 (3,6)
PF3D7_1437900	Heat shock protein 40	HSP40	Cytosolic chaperone	2.33%	5	11 (5,6)
PF3D7_1010700	Dolichyl-phosphate-mannose-protein mannosyltransferase, putative	ALG2	CUL3 ubiquitin ligase adaptor protein, with role in dolichol metabolism	2.32%	6	11 (5,6)
PF3D7_0520900	Adenosylhomocysteinase	SAHH	Methionine metabolism	2.15%	3	6 (3,3)
PF3D7_1026800	40S ribosomal protein S2	RPS2	Small ribosomal subunit	1.76%	6	12 (5,7)
PF3D7_1444800	Fructose-bisphosphate aldolase	FBPA	Glycolysis	1.72%	3	6 (3,3)
PF3D7_0105200	RAP domain-containing protein	--	Altered transcription following inhibition of polyamine and methionine metabolism enzymes	1.51%	5	12 (5,7)
PF3D7_0915400	ATP-dependent 6-phosphofructokinase	PFK9	Cytoplasm	1.50%	3	5 (2,3)
PF3D7_0822600	Protein transport protein Sec23	SEC23	COPII mediated vesicular transport	1.50%	4	7 (3,4)
PF3D7_0934500	V-type proton ATPase subunit E, putative	--	Ca^2+^ homeostasis	1.49%	5	11 (5,6)
PF3D7_0626800	Pyruvate kinase	PyrK	Glycolysis / Interaction with HDAC1	1.49%	4	6 (3,3)
PF3D7_1008700	Tubulin beta chain	--	Microtubules	1.40%	4	8 (4,4)
PF3D7_1412500	Actin II	ACT2	Actin filaments	1.38%	4	9 (4,5)
PF3D7_0929200	RNA-binding protein, putative	--	RNA-binding protein	1.27%	3	6 (2,4)
PF3D7_0927300	Fumarate hydratase, putative	FH	Mitochondrial TCA cycle	1.24%	6	9 (4,5)
PF3D7_0623500	Superoxide dismutase [Fe]	SOD2	Mitochondrial antioxidant system	1.21%	4	8 (4,4)
PF3D7_0820700	2-oxoglutarate dehydrogenase E1 component	KDH	Mitochondrial TCA cycle	1.20%	3	7 (3,4)
PF3D7_1037100	Pyruvate kinase 2	PyKII	Apicoplast / Isoprenoid metobolismmetabolism	1.19%	4	8 (4,4)
PF3D7_0608800	Ornithine aminotransferase	OAT	Ornithine metabolism	1.18%	3	5 (2,3)
PF3D7_1302100	Gamete antigen 27/25	Pfs27/25	Early marker of gametocyte development	1.13%	4	8 (3,5)
PF3D7_1327800	Ribose-phosphate pyrophosphokinase, putative	--	Pentose phosphate cycle	1.12%	5	7 (2,5)
PF3D7_0823900	Dicarboxylate/tricarboxylate carrier	DTC	Mitochondrial antioxidant system, TCA cycle	1.07%	4	9 (4,5)
PF3D7_1215000	Thioredoxin peroxidase 2	Trx-Px2	Mitochondrial antioxidant system	1.06%	4	7 (2,5)
PF3D7_1472600	Protein disulfide-isomerase	PDI-14	Oxidative protein folding in the ER, component of chaperone complexes that interact with BiP	1.06%	4	7 (3,4)
PF3D7_0720400	Ferrodoxin NADP+ reductase	--	Mitochondrial iron-sulfur protein biogenesis	1.05%	3	5 (2,3)
PF3D7_1468700	Eukaryotic initiation factor 4A	eIF4A	Eukaryotic translation initiation factor 4F complex	1.02%	4	7 (3,4)
PF3D7_0722200	Rhoptry-associated leucine zipper-like protein 1	RALP1	Rhoptry neck protein	0.99%	4	6 (4,2)
PF3D7_1410600	Eukaryotic translation initiation factor 2 subunit gamma, putative	eIF2g	Eukaryotic translation initiation factor 2 complex	0.92%	3	7 (3,4)
PF3D7_1338200	60S ribosomal protein L6-2, putative	--	Large ribosomal subunit	0.92%	4	9 (4,5)
PF3D7_1320000	Golgi protein 1	GP1	ER-Golgi translocation and quality control	0.89%	3	6 (3,3)
PF3D7_1437700	Succinyl-CoA ligase, putative	--	Mitochondrial TCA cycle	0.86%	4	6 (1,5)
PF3D7_0829200	Prohibitin, putative	PHB1	Mitochondrial protein degradation	0.83%	4	9 (4,5)
PF3D7_0621200	Pyridoxine biosynthesis protein PDX1	PDX1	Vitamin B6 synthesis	0.83%	3	6 (3,3)
PF3D7_1330600	Elongation factor Tu, putative	--	Mitochondrial protein translation	0.83%	4	8 (3,5)
PF3D7_0616800	Malate:quinone oxidoreductase, putative	MQO	Mitochondrial TCA cycle, ETC	0.81%	4	7 (2,5)
PF3D7_0624000	Hexokinase	HK	Glycolysis	0.79%	3	6 (3,3)
PF3D7_0920800	Inosine-5'-monophosphate dehydrogenase	IMPDH	Purine metabolism	0.78%	3	5 (2,3)
PF3D7_0511800	Inositol-3-phosphate synthase	INO1	Inositol phosphate metabolism	0.73%	3	5 (3,2)
PF3D7_0727200	Cysteine desulfurase, putative	NFS	Mitochondrial iron-sulfur protein biogenesis	0.72%	3	5 (1,4)
PF3D7_0813900	40S ribosomal protein S16, putative	--	Ribosome	0.70%	3	9 (3,6)
PF3D7_1108400	Casein kinase 2, alpha subunit	CK2a	Cytoplasm, nucleus, calcium dependent protein kinase (CK2 complex)	0.69%	4	9 (4,5)
PF3D7_1115600	Peptidyl-prolyl cis-trans isomerase	CYP19B	Oxidative protein folding in the ER, component of chaperone complexes that interact with BiP	0.69%	3	5 (2,3)
PF3D7_1320800	Dihydrolipoyllysine-residue succinyltransferase component of 2-oxoglutarate dehydrogenase complex	--	Mitochondrial TCA cycle	0.68%	4	7 (3,4)
PF3D7_1408600	40S ribosomal protein S8e, putative	--	Ribosome	0.68%	3	6 (2,4)
PF3D7_0816600	Chaperone protein ClpB	ClpB	Chaperone-assisted protein folding (apicoplast and/or mitochondrion)	0.68%	4	8 (3,5)
PF3D7_1133400	Apical membrane antigen 1	AMA1	Invasion molecule	0.67%	3	6 (2,4)
PF3D7_0709700	Prodrug activation and resistance esterase	PARE	Cytosolic esterase, putative lipase	0.65%	5	9 (4,5)
PF3D7_0401800	*Plasmodium* exported protein (PHISTb), unknown function	PfD80	Maurer's cleft exported protein	0.64%	4	8 (4,4)
PF3D7_0316800	40S ribosomal protein S15A, putative	--	Ribosome	0.63%	5	9 (5,4)
PF3D7_1465900	40S ribosomal protein S3	--	Ribosome	0.62%	3	6 (2,4)
PF3D7_0106800	Ras-related protein Rab5C	RAB5C	Intracellular traffic / Endocytosis	0.61%	3	6 (3,3)
PF3D7_1025300	Conserved *Plasmodium* protein, unknown function	--	No known or predicted function	0.60%	3	6 (2,4)
PF3D7_1136300	Tudor staphylococcal nuclease	TSN	mRNA splicing	0.60%	4	7 (2,5)
PF3D7_1361100	Protein transport protein Sec24A	SEC24A	COPII mediated vesicular transport	0.59%	4	6 (2,4)
PF3D7_1439400	Cytochrome bc1 complex subunit Rieske, putative	--	Mitochondrial cytochrome bc1 complex (ETC)	0.55%	4	7 (3,4)
PF3D7_1452000	Rhoptry neck protein 2	RON2	Invasion molecule	0.54%	3	5 (2,3)
PF3D7_1212500	Glycerol-3-phosphate 1-O-acyltransferase	GAT	ER membrane protein / Glycerolipid synthesis	0.54%	4	8 (3,5)
PF3D7_0922500	Phosphoglycerate kinase	PGK	Glycolysis	0.50%	4	5 (2,3)
PF3D7_1008400	26S proteasome AAA-ATPase subunit RPT2, putative	RPT2	Proteasome	0.49%	4	6 (3,3)
PF3D7_0416800	Small GTP-binding protein Sar1	SAR1	COPII mediated vesicular transport	0.49%	4	7 (4,3)
PF3D7_0810600	ATP-dependent RNA helicase DBP1, putative	DBP1	Helicase	0.48%	3	6 (2,4)
PF3D7_1145400	Dynamin-like protein	DYN1	Clathrin-mediated vesicular transport	0.45%	3	6 (3,3)
PF3D7_0309600	60S acidic ribosomal protein P2	PfP2	Ribosome	0.43%	4	5 (3,2)
PF3D7_0112200	Multidrug resistance-associated protein 1	MRP1	Plasma membrane component / glutathione and redox metabolism	0.41%	3	6 (3,3)
PF3D7_0504600	2-oxoisovalerate dehydrogenase subunit beta, mitochondrial, putative	BCKDHB	Mitochondrial TCA cycle	0.41%	3	6 (3,3)
PF3D7_1306400	26S proteasome AAA-ATPase subunit RPT4, putative	RPT4	Proteasome	0.36%	3	5 (2,3)
PF3D7_0524000	Karyopherin beta	KASb	Import and export through the nuclear pore	0.35%	4	6 (3,3)
PF3D7_0702500	*Plasmodium* exported protein, unknown function	--	Maurer's cleft exported protein	0.33%	3	7 (3,4)
PF3D7_1361900	Proliferating cell nuclear antigen 1	PCNA1	DNA replication and repair	0.32%	3	6 (2,4)
PF3D7_0507100	60S ribosomal protein L4	RPL4	Ribosome	0.30%	3	6 (3,3)
PF3D7_1247400	Peptidyl-prolyl cis-trans isomerase FKBP35	FKBP35	Cytoplasm, nucleus, centrosome	0.30%	3	5 (1,4)
PF3D7_0213700	Conserved *Plasmodium* protein, unknown function	--	No known or predicted function	0.30%	3	5 (3,2)
PF3D7_0217900	Conserved *Plasmodium* protein, unknown function	--	Putative thioesterase	0.29%	3	5 (1,4)
PF3D7_0205900	26S proteasome regulatory subunit RPN1, putative	RPN1	Proteasome	0.29%	3	5 (3,2)
PF3D7_0719700	40S ribosomal protein S10, putative	--	Ribosome	0.28%	3	5 (1,4)
PF3D7_1105000	Histone H4	H4	Nucleus	0.27%	4	5 (3,2)
PF3D7_0413500	Phosphoglucomutase 2, putative	PGM2	Glycolysis	0.25%	3	6 (3,3)
PF3D7_0520000	40S ribosomal protein S9, putative	--	Ribosome	0.23%	3	7 (4,3)
PF3D7_1129200	26S proteasome regulatory subunit RPN7, putative	RPN7	Proteasome	0.22%	3	5 (3,2)

^1^A summary of these experiments can be found in S2 Table.

^2^The 13 samples were derived from Cam3.II^WT^ (4), Cam3.II^R539T^ (4), Cam3.II^C580Y^ (2), CamWT^WT^ (2), and CamWT^C580Y^ (1).

After K13, the most abundant protein in our co-IPs was S-adenosylmethionine (SAM) synthetase (also known as methionine adenosyltransferase), a redox-regulated enzyme that produces the methyl donor S-adenosylmethionine used in methylation reactions of multiple substrates including nucleic acids, proteins, phospholipids and amines [36]. Adenosylhomocysteinase (also known as adenosylhomocysteine hydrolase), another enzyme involved in the methionine metabolism pathway that produces SAM, was also in the top six most abundant proteins. Phosphoglycerate mutase 1 (PGM1) was the second most abundant protein, with phosphoglucomutase 2 (PGM2) being less abundant. Intriguingly PGM5, the mammalian homolog of PGM2, tethers the K13 ortholog Keap1 to the mitochondria [37,38]). PGM1 is annotated as being involved in parasite glycolysis, a pathway implicated with several other immunoprecipitated proteins. The receptor for activated c kinase (RACK), a cytosolic multi-functional scaffolding protein, was also abundant.

Consistent with our data that localized K13 to intracellular foci, we reproducibly co-immunoprecipitated K13 with several proteins involved in vesicular trafficking (**Table 2** and **S2 Table**). These included multiple members of the Rab family of GTPases, namely Rab1A, Rab1B, Rab5C, Rab6, Rab7, Rab11B, and Rab18B, which function as regulators of vesicular trafficking and endocytosis in eukaryotes [39]. By applying an overrepresentation test in the PANTHER classification system for biological processes, we observed an 18-fold enrichment in Rab proteins, with a *p* value of 1×10^−6^ (**S3 Table;** http://pantherdb.org; [40,41]). K13-specific mAbs also co-immunoprecipitated Sar1, which assists in COPII coat assembly, and Sec23 and Sec24a, which form a heterodimer associated with the COPII vesicle coat that surrounds transport vesicles budding from the ER. In *P*. *falciparum*, Sec24a has been localized to transitional ER sites where it mediates the capture of COPII vesicle cargo [42]. Our co-IP studies also identified other ER-associated proteins, including Sec61, part of the ER-Sec61 translocon complex [43,44], the luminal protein disulfide isomerase (PDI-14), and two peptidyl-prolyl cis-trans isomerases (CYP19B and FKBP35) that catalyze protein folding in the ER.

Multiple members of the eukaryotic translation machinery were also identified, including protein components of the 40S and 60S ribosomes and several translation initiation or elongation factors (EIF1α, eEF2, eIF2α, eIF4A), as well as several nucleic acid-binding proteins (including SR1 and SR4). Multiple components of the 19S regulatory particle of the 26S proteasome were observed, notably RPT2, RPT4, RPN1, and RPN7. The 26S proteasome likely has a role in degrading ART-damaged proteins, supporting a potential role for K13 as a ubiquitin ligase adaptor protein that could help deliver polyubiquinated proteins for proteasome-mediated degradation [45].

We also identified several proteins that localize to the DV, wherein host hemoglobin is degraded leading to the release of reactive heme. These include the hemoglobin-processing enzyme plasmepsin II, and the membrane proteins PfCRT and falcilysin, with the latter also being involved in transit peptide degradation in the apicoplast and mitochondria [46].

Unexpectedly, several proteins were also detected that are known or predicted to localize to mitochondria. These include the Rieske protein that is part of the cytochrome bc1 complex, as well as prohibitin 1 that is implicated in mitochondrial morphogenesis and that is a possible regulator of mitochondrial membrane potential [47]. Components of the TCA cycle (including fumarate hydratase, the 2-oxoglutarate dehydrogenase E1 component, a dicarboxylate/tricarboxylate carrier, succinyl-CoA ligase, malate:quinone oxidoreductase, and the 2-oxoisovalerate dehydrogenase beta subunit) were also observed. We also identified factors thought to be involved in mitochondrial translation (elongation factor Tu), protein degradation (the ATP-dependent protease subunit ClpQ, the ATP-dependent zinc metalloprotease FTSH 1, and the mitochondrial-processing peptidase alpha subunit), iron-sulfur protein biogenesis (ferrodoxin NADP+ reductase and cysteine desulfurase), as well as two putative mitochondrial chaperones (HPS40 and CPN20).

Other potential K13-interacting proteins associated with the mitochondria included the ATP synthase F1 alpha subunit (involved in mitochondrial energy metabolism), mitochondrial matrix protein 33, and a putative dynamin (involved in mitochondrial fission). Finally, several proteins were associated with the mitochondrial antioxidant system, including superoxide dismutase, thioredoxin peroxidase 2, isocitrate dehydrogenase, the succinyl-CoA synthetase alpha subunit, a lipoamide acyltransferase, and the glutathione peroxidase-like thioredoxin peroxidase. PANTHER overrepresentation analysis focusing on cellular components revealed a 15-fold enrichment in mitochondrial proteins, with a *p* value of 4×10^−4^ (**S4 Table**).

In reviewing putative functional features of proteins in our K13 co-IP list (**Table 2** and **S2 Table**), we observed an apparent enrichment of proteins known to undergo post-translational modifications, specifically palmitoylation (73 proteins), glutathionylation (59 proteins), and S-nitrosylation (53 proteins). In comparison, 409, 493 and 319 proteins with these respective modifications were identified from the total asexual blood stage proteome (comprising over 4,800 proteins based on detected expression ([48–51]; http://mpmp.huji.ac.il)). This corresponds to an estimated three to five-fold enrichment in these proteins among putative K13-interacting partners.

### K13 partially co-localizes with proteins involved in vesicular trafficking and cytostomes

To further interrogate the putative interactors identified by our co-IP studies, we performed quantitative co-localization studies using our K13 mAbs or K13 tagged lines combined with other epitope-tagged lines or primary antibodies to proteins of interest. Initial experiments focused on the Rab GTPase family and the Sec23/24 heterodimer. For these studies, we prepared highly synchronized early ring-stage parasites (0–3 hpi) and pulsed these for 6h with 700 nM DHA. Control cultures were mock-treated with DMSO vehicle. Parasites were harvested at various time points post drug pulse and subsequently fixed and stained for microscopic analysis (**S3 Fig**). To increase throughput and reproducibility we developed a quantitative analysis pipeline in collaboration with Nikon software engineers for image processing and determination of PCC values (listed in **S5 Table**).

**Fig 2:**
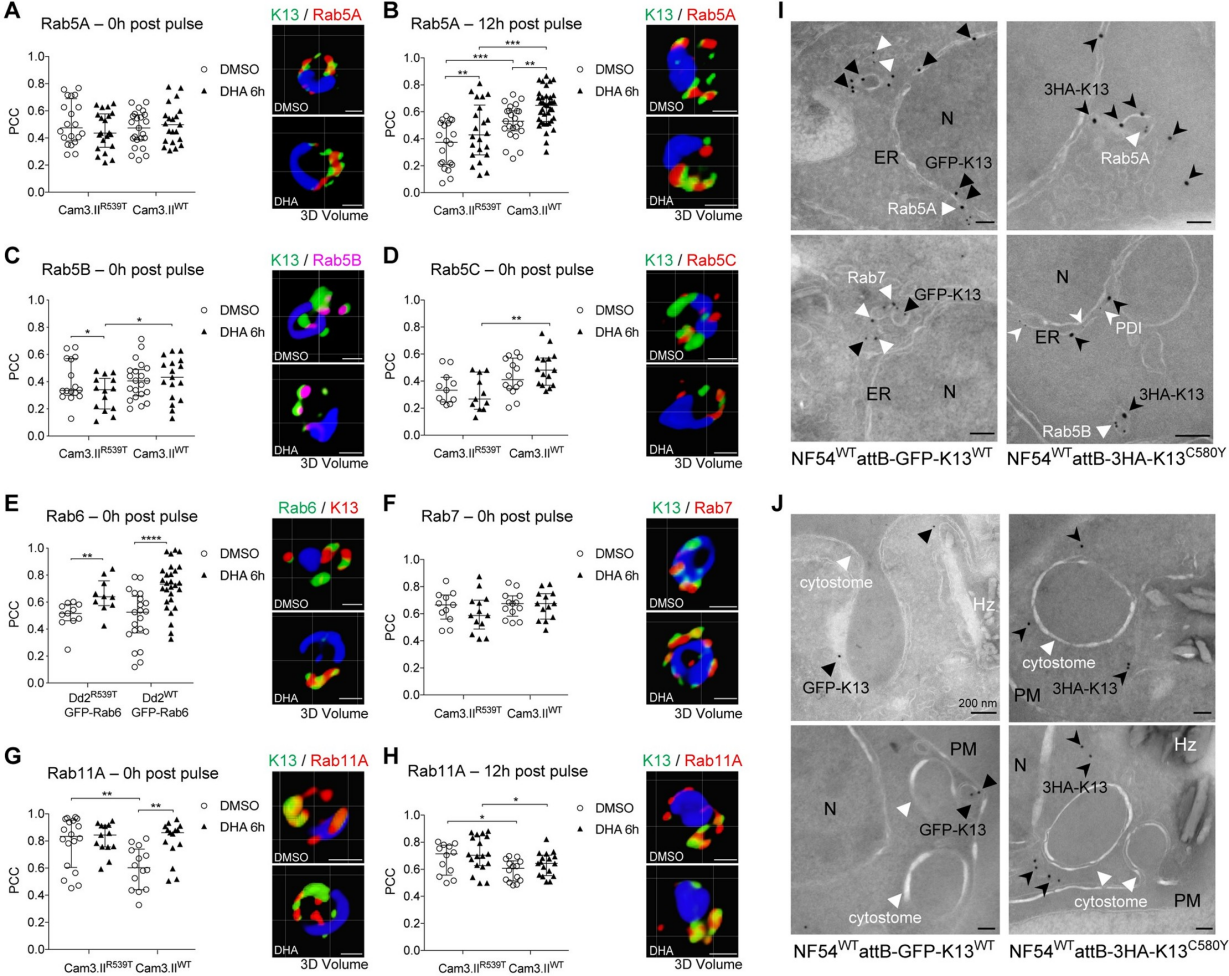
K13 partially co-localizes with vesicular transport proteins and sites adjacent to cytostomes. **(A-H)** PCC values quantifying the degree of spatial co-localization between K13 and **(A, B)** Rab5A, **(C)** Rab5B, **(D)** Rab5C, **(E)** Rab6, **(F)** Rab7, and **(G, H)** Rab11A, with accompanying representative 3D volume reconstructions of IFA images. Assays were conducted using Cam3.II^R539T^ or Cam3.II^WT^ parasites, except in the case of Rab6, where Dd2^R539T^ or Dd2^WT^ parasite lines expressing GFP-Rab6 were employed. Samples were collected either directly following a 6h 700 nM pulse of DHA **(A, C-G**; denoted 0h**)** or 12h post pulse **(B, H)**. DMSO was used as a vehicle control. K13 was labeled using the E3 mAb (green). Rab proteins were labeled with specific antibodies (red) or, in the case of Rab6, anti-GFP (green, with K13 this time in red). PCC values were calculated from the fluorescence intensity correlations of Alexa Fluor 488 and either Alexa Fluor 594 or Alexa Fluor 647. For PCC plots, each dot represents an individual parasitized RBC. Horizontal lines represent the median with interquartile range. Two-tailed unpaired *t* tests were used to calculate *p* values. *p<0.05; **p<0.01; ***p<0.001; ****p<0.0001. Scale bars: 1 μm. **(I)** IEM images of trophozoite-stage NF54^WT^attB-GFP-K13^WT^ (left) or NF54^WT^attB-3HA-K13^C580Y^ (right) parasites co-stained with anti-GFP or anti-HA, respectively, and anti-Rab5A (two upper panels), anti-Rab7 (bottom left) or anti-Rab5B plus anti-PDI (bottom right). Secondary antibodies were conjugated to colloidal gold particles of different sizes. Arrowheads highlight locations of interest. ER, endoplasmic reticulum; N, nucleus. Scale bars: 100 nm. **(J)** IEM images of NF54^WT^attB-GFP-K13^WT^ (left) or NF54^WT^attB-3HA-K13^C580Y^ (right) trophozoites stained with anti-GFP or anti-HA, respectively. Arrowheads highlight locations of interest. Hz, hemozoin; N, nucleus; PM, plasma membrane. Scale bars: 100 nm unless otherwise indicated.

We first assayed the Cam3.II^WT^ and Cam3.II^R539T^ lines with antibodies to Rab5A, 5B, or 5C, along with our K13-specific E3 mAb. Samples were collected immediately post drug treatment (0h time point, ~6 hpi), and, in the case of Rab5A, also at 12h post pulse (~18 hpi). IFAs with anti-K13 and anti-Rab5A antibodies showed an intermediate degree of spatial association between the two proteins immediately (0h) post pulse in Cam3.II^WT^ and Cam3.II^R539T^, with median PCC values for both lines centering around 0.5 (**Fig 2A** and **S4A Fig**). At this time point, we observed no effect of DHA treatment on PCC values in either line. At 12h post pulse, however, we observed a statistically significant increase in PCC values for both mutant and WT parasites in DHA-treated cultures as compared to mock (DMSO) treatment (**Fig 2B** and **S4B Fig**). At this 12h time point with DHA-treated parasites, the PCC values for K13 and Rab5A were significantly higher for K13 WT parasites compared with their mutant counterparts (median 0.65 vs 0.43; *p*<0.001; **Fig 2B** and **S5 Table**).

The putative K13 associations with Rab5B or Rab5C were also examined immediately post DHA or DMSO treatment. Median PCC values for K13 and Rab5B or Rab5C were slightly lower than for Rab5A in both Cam3.II^WT^ and Cam3.II^R539T^ parasites (**Fig 2C and 2D** and **S4A Fig**). For both Rab5B and 5C, a slightly stronger association with K13 was observed in WT parasites compared with the mutants, a trend that became statistically significant upon DHA treatment (*p*<0.05 and *p*<0.01, respectively). With all three Rab proteins, there was a trend towards slightly lower PCC values immediately post DHA treatment (0h time point) in the K13 mutant line and slightly increased PCC values in the K13 WT line. These data suggest decreased levels of endocytosis in K13 mutant parasites following DHA exposure.

We subsequently assessed the spatial association between K13 and Rab6, a trans-Golgi marker known to direct exocytic vesicles to the plasma membrane in mammalian cells [52]. These experiments were conducted using Dd2 K13 WT or R539T parasites expressing Rab6-GFP episomally. PCC values were moderately high in both DMSO-treated lines, and were significantly increased in both lines directly post DHA pulse, with the highest levels of association (median 0.73) observed in DHA-treated K13 WT parasites (**Fig 2E**, **S4C Fig** and **S5 Table**).

We continued our IFA analyses with the late endosome marker Rab7 and the post Golgi marker Rab11A, using the Cam3.II^WT^ and Cam3.II^R539T^ lines and antibodies to K13 and the two Rab proteins. For Rab7 we observed relatively high median PCC values, centering around 0.6, regardless of *K13* allele status. These remained unchanged immediately post DHA treatment (0h time point; **Fig 2F** and **S4D Fig**). For Rab11A we measured high PCC values for co-localization with K13 in Cam3.II^R539T^ parasites for all time points and conditions examined, including 0h and 12h post DHA and DMSO treatments (median 0.70–0.84; **Fig 2G and 2H**, **S4D and S4E Fig**, and **S5 Table**). In contrast, median PCC values for K13 and Rab11A were significantly lower in WT parasites than in mutant parasites at both time points, in particular for DMSO-treated cultures (0.60–0.61; **S5 Table**). A slight but nonetheless significant increase was observed in K13 and Rab11A association in WT parasites immediately after DHA treatment (0h time point; *p*<0.01), bringing PCC values to the level of K13 mutant parasites, but these values dropped again at 12h post treatment (**Fig 2G and 2H**).

To extend these co-localization studies, we also performed ultrastructural analyses via IEM. These studies used the NF54^WT^attB-GFP-K13^WT^ and NF54^WT^attB-3HA-K13^C580Y^ lines, which were stained with anti-GFP or anti-HA colloidal gold-conjugated primary antibodies. Co-staining with antibodies specific for Rab proteins localized K13 (black arrowheads) to sites either within or adjacent to vesicles containing Rab5A, Rab5B or Rab7 (white arrowheads; **Fig 2I** and **S4F Fig**). In some IEM images we also observed K13 near cytostomes (**Fig 2J**).

Our co-IP data suggested that K13 might also partially associate with the transitional ER marker Sec24a, involved in vesicle budding from the ER. To examine this further, we tested our anti-K13 E3 mAb on a Sec24a-GFP expressing parasite line that harbors WT K13 [42]. Quantitative IFA analyses revealed intermediate to high median PCC values (0.60–0.67) for Sec24a and K13, with no significant changes upon DHA treatment (**S4G and S4H Fig**).

### K13 partially co-localizes with the ER chaperone BiP but not with the cis-Golgi marker ERD2

**Fig 3:**
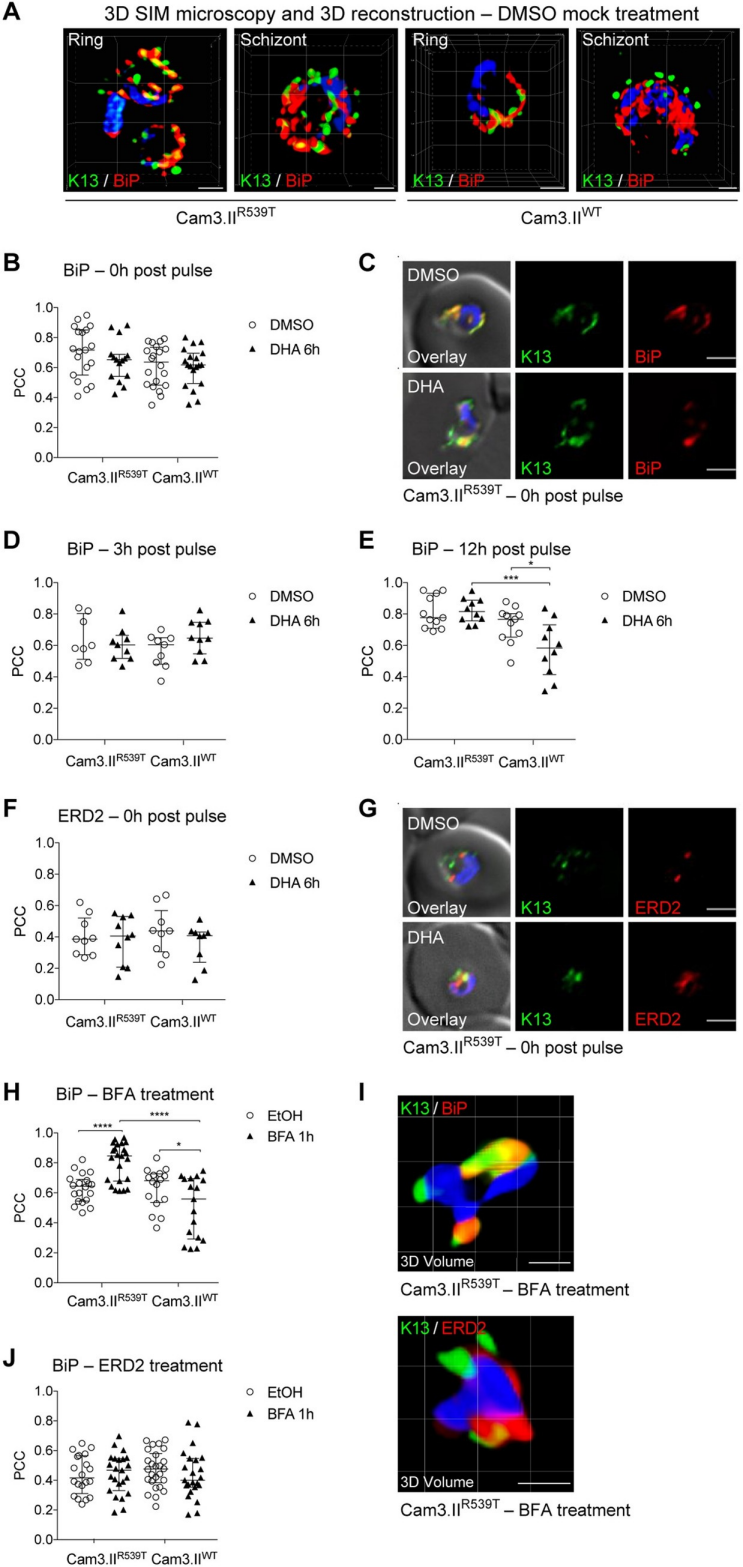
K13 shows substantial co-localization with the ER chaperone BiP but not with the cis-Golgi marker ERD2. **(A)** 3D SIM microscopy images showing Cam3.II^R539T^ or Cam3.II^WT^ parasites co-stained with the K13 E3 mAb (green) and antibodies to the ER chaperone BiP (red). SIM: structured illumination microscopy. Scale bars: 1 μm. **(B, C)** PCC values quantifying co-localization between K13 and BiP at 0h post DHA (6h, 700 nM) pulse, alongside representative IFA images. DMSO was used as a vehicle control. Assays were conducted using the Cam3.II^R539T^ and Cam3.II^WT^ lines. Parasites were co-stained with the K13 E3 mAb and antibodies to BiP. PCC values were calculated and statistics performed as in **Fig 2**. Scale bars: 2 μm. **(D, E)** PCC values for co-localization of K13 and BiP at **(D)** 3h or **(E)** 12h post drug pulse. Assays were conducted and PCC values were calculated as in **(B)**. **(F, G)** PCC values quantifying co-localization between K13 and ERD2 at 0h post DHA pulse, alongside representative IFA images. Parasites were co-stained with the K13 E3 mAb and antibodies to ERD2. Assays were otherwise conducted as in **(B)**. Scale bars: 2 μm. **(H-J)** PCC values for co-localization of K13 and BiP or ERD2 following a 1h treatment with Brefeldin A (BFA; 5 μg/ml). EtOH was used as a vehicle control. Assays were conducted using the Cam3.II^R539T^ and Cam3.II^WT^ lines. **(I)** 3D volume reconstruction of deconvolved Z-stacks (15 image stacks, step size of 0.2 μm) of Cam3.II^R539T^ ring-stage parasites treated with BFA and co-stained with anti-K13 mAb E3 and anti-BiP (top) or anti-ERD2 (bottom). Scale bars: 1 μm.

Given that K13 also co-immunoprecipitated a number of ER-associated proteins (**Table 2** and **S2 Table**), we performed imaging analyses using markers for the ER and cis-Golgi, namely BiP and ERD2, respectively. To test for K13 and BiP spatial association, we applied high-resolution 3D structured illumination microscopy to ring- and schizont-stage parasites. These assays employed the Cam3.II K13 WT and R539T mutant lines, which were stained with anti-K13 and anti-BiP antibodies (**Fig 3A**). In the ring stages, both mutant and WT K13 proteins localized to foci associated with the BiP-labeled ER, whereas in the schizont stages only R539T K13 showed evidence of a close spatial association with BiP. This association was supported via widefield immunofluorescence microscopy with trophozoites (**S5A Fig**). Close proximity between K13 and the ER was also observed by IEM studies with NF54^WT^attB-GFP-K13^WT^ parasites labeled with anti-BiP and anti-GFP antibodies (**S5B Fig**), as well as our previous IEM studies with triple labeling of K13, Rab5B and the ER chaperone PDI (white arrowheads; **Fig 2I** and **S4F Fig**).

We next assessed the degree of co-localization between K13 and BiP in DMSO- and DHA- treated Cam3.II^WT^ or Cam3.II^R539T^ samples throughout the first half of the IDC, with time points taken at 0h, 3h, 12h and 24h post treatment (**S3 Fig**). In DMSO-treated samples we observed a non-significant trend towards lower PCC values in K13 WT samples as compared to mutant samples across all time points tested (**Fig 3B–3E** and **S5C Fig**). At the 0h time point, for example, PCC values for K13 and BiP averaged 0.72 and 0.64 for Cam3.II^R539T^ and Cam3.II^WT^ respectively (**S5 Table**). By comparison, a very recent study using K13-specfic polyclonal antiserum reported a PCC value of 0.58 between WT K13 and BiP [53]. Interestingly, following DHA treatment, mutant and WT parasites showed significant differences at 12h post drug pulse (**Fig 3E** and **S5C Fig**). Whereas PCC values for WT parasites dropped significantly post DHA treatment, PCC values for K13 and BiP in R539T parasites were highest post DHA treatment at this time point (median 0.82; **S5 Table**).

In light of the proximity between the cis-Golgi and sites of vesicle budding from the ER, we compared association coefficients obtained with BiP to those measured using ERD2, a marker of the cis-Golgi, using the same lines. In contrast to the high PCC values observed for K13 and BiP in Cam3.II^WT^ or Cam3.II^R539T^, for K13 and ERD2 we measured moderate to low PCC values regardless of the condition and parasite line tested (median 0.39–0.4; **Fig 3F and 3G**, **S5D Fig** and **S5 Table**). These data argue against K13 being present in the cis-Golgi.

To assess whether K13 localization was affected by blocking ER-to-Golgi transport, we exposed parasites to the fungal metabolite brefeldin A (BFA). This agent perturbs secretory traffic in parasites, resulting in retention of secreted proteins within the ER [54]. In these experiments, we treated Cam3.II K13 WT and mutant ring-stage parasites (0–3 hpi) with BFA or vehicle control (EtOH) for 1h before harvesting and co-staining with anti-K13 and antibodies to either BiP or ERD2. After the BFA pulse we observed a significantly higher association between K13 and BiP in K13 mutant parasites, as compared with the ethanol (EtOH) mock-treated population (median 0.85 vs 0.64, respectively; **S5 Table**). By comparison, we measured significantly lower PCC values for K13 and BiP in BFA-pulsed vs mock-treated K13 WT parasites (median 0.68 vs 0.56; **Fig 3F** and **S5 Table**). In contrast, PCC values for K13 and ERD2 remained moderate regardless of the treatment (BFA or mock; **Fig 3J**) and were equivalent to those observed in the DHA and DMSO treatments in both lines (**Fig 3F**). The increased association between BiP and mutant K13 upon BFA treatment was further illustrated for Cam3.II^R539T^ in 3D volume reconstructions of K13- and BiP-labeled ring-stage parasites (**Fig 3I**).

### K13 shows an elevated association with mitochondria post dihydroartemisinin pulse

**Fig 4:**
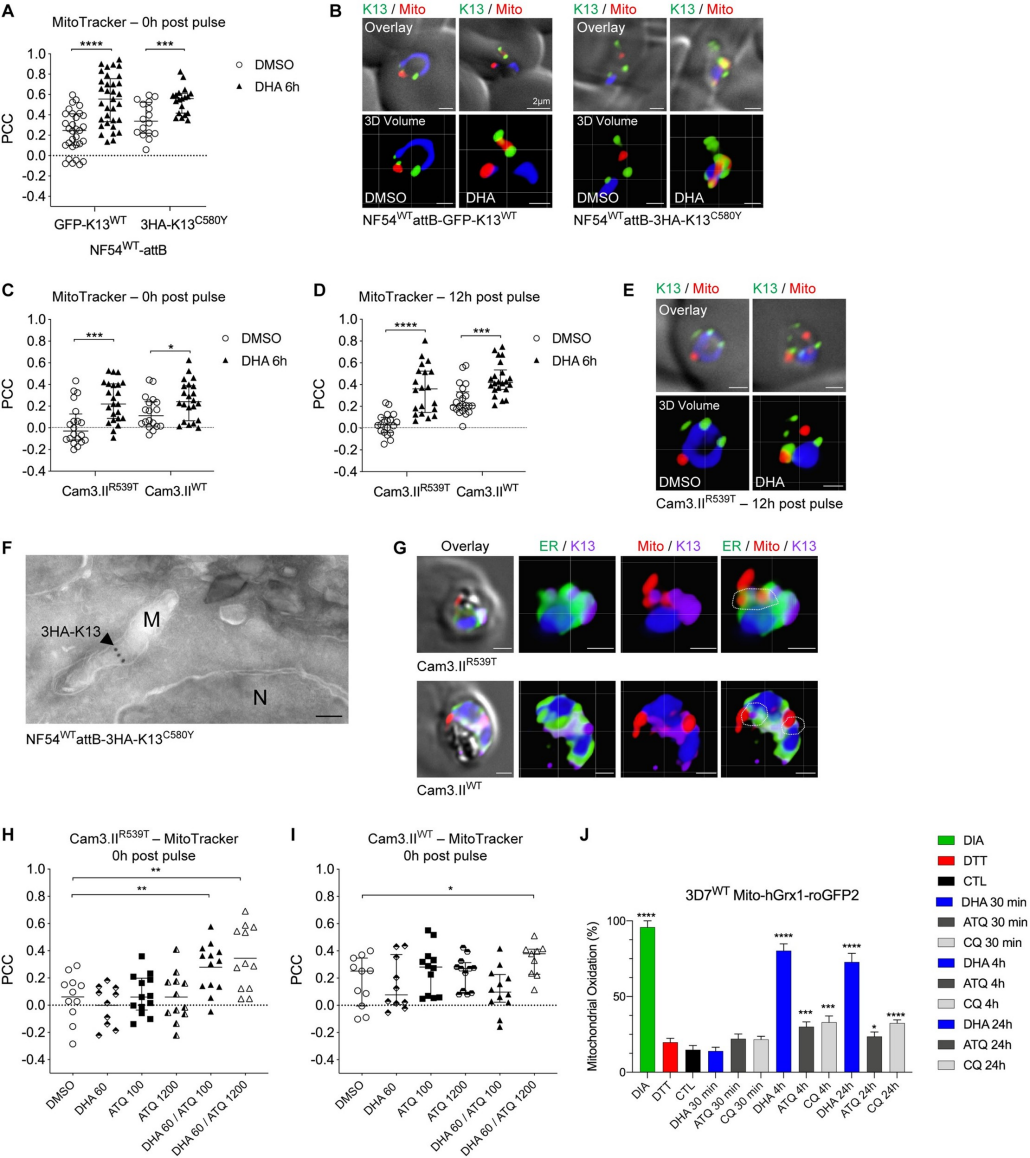
K13 shows an increased association with the mitochondria post DHA pulse. **(A)** PCC values for the association of K13 with parasite mitochondria in NF54 ^WT^attB-GFP-K13^WT^ or NF54^WT^attB-3HA-K13^C580Y^ ring-stage parasites co-stained with MitoTracker Deep Red and anti-GFP antibodies. Samples were collected directly post DHA pulse (6h, 700 nM). DMSO was used as a vehicle control. **(B)** Representative fluorescence microscopy images and 3D reconstructions of NF54^WT^-attB-GFP-K13^WT^ (left) or NF54^WT^attB-3HA-K13^C580Y^ (right) ring-stage parasites treated and stained as in **(A)**. Scale bars: 1 μm unless otherwise indicated. **(C, D)** PCC values for the association of K13 with the mitochondria in Cam3.II^R539T^ or Cam3.II^WT^ ring-stage parasites co-stained with MitoTracker and the K13 mAb E3. Samples were collected either **(C)** immediately post DHA pulse (6h, 700 nM) or DMSO mock treatment or **(D)** 12h post pulse. **(E)** Representative fluorescence microscopy images and 3D reconstructions of Cam3.II^R539T^ ring-stage parasites 12h post DHA pulse or DMSO mock treatment. Scale bars: 1 μm. **(F)** Representative IEM images of NF54^WT^attB-3HA-K13^C580Y^ trophozoites treated with DHA (9 nM for 3h) or a DMSO vehicle control and stained with anti-HA antibodies to detect K13. Arrowhead highlights location of interest. M, mitochondria; N, nucleus. Scale bar: 100 nm. **(G)** 3D volume reconstruction of untreated late Cam3.II^R539T^ and Cam3.II^WT^ trophozoites triply stained with MitoTracker, anti-BIP (ER, green) and anti-K13 E3 (purple). White dotted outlines indicate spatial overlap between the three labels. Scale bars: 1 μm. **(H, I)** PCC values for the association of K13 with mitochondria in **(H)** Cam3.II^R539T^ or **(I)** Cam3.II^WT^ ring-stage parasites treated for 4h with 60 nM DHA, or 100 nM or 1200 nM ATQ, or combinations thereof. Samples were co-stained with MitoTracker and the K13 mAb E3. **(J)** Percent mitochondrial oxidation (OxD; see Methods) measured in the 3D7^WT^ Mito-hGrx1-roGFP2 reporter line. Parasites were treated with DHA, ATQ, CQ, a known oxidizing agent (DIA), a known reducing agent (DTT), or vehicle control (denoted CTL). Parasites were exposed for 30 min to 100 μM drug concentrations, for 4h to 5 μM drug, or for 24h to 50 nM drug. Experiments were performed on three independent occasions, with 10 parasites per experiment examined using confocal laser scanning microscopy. Results are presented as means ± SEM. Significance was calculated using two-tailed, unpaired *t* tests comparing drug-treated with mock-treated parasites. *p<0.05; **p<0.01; ***p<0.001; ****p<0.0001. ATQ, atovaquone; CQ, chloroquine; DHA, dihydroartemisinin; DIA, diamide; DTT, 1,4-dithiothreitol.

To explore the link between K13 and the mitochondrial proteins observed in our co-IP studies (**Table 2** and **S2 Table**), we quantified the degree of co-localization between K13 and mitochondria using our anti-K13 antibodies together with the live mitochondrial dye MitoTracker Deep Red. PCC values were determined either immediately (0h) or 12h post drug pulse (i.e. 6h of DHA or DMSO vehicle). These assays used the NF54^WT^attB-GFP-K13^WT^ and NF54^WT^attB-3HA-K13^C580Y^ lines, as well as the isogenic Cam3.II K13 WT and R539T pair. Median PCC values for mock-treated parasites were low (ranging from -0.03 to 0.25), as shown in representative images (**Fig 4A–4E** and **S6 Table**). After DHA treatment we observed significantly increased associations between the mitochondria and K13 at both time points in all parasite lines tested, particularly at the 12h time point in NF54^WT^attB-GFP-K13^WT^ parasites (median PCC value 0.84 in DHA-treated parasites vs. 0.44 in mock-treated; **S6A Fig** and **S6 Table**). For the isogenic Cam3.II^R539T^ and Cam3.II^WT^ lines, a greater increase was observed in the mutant parasites as compared to their K13 WT counterparts, especially 12h post drug treatment (**Fig 4D**). The increased co-localization observed between parasite mitochondria and K13 following DHA treatment was not detected when MitoTracker-labeled parasites were co-stained with Rab5A, Rabb11A, TRiC or ERD2 (**S6B–S6E Fig**).

IEM studies of NF54^WT^attB-3HA-K13^C580Y^ parasites stained with colloidal gold-labeled anti-HA antibodies revealed some K13 labeling within parasite mitochondria (**Fig 4F**). Because mitochondria associate with the ER at specialized membrane contact sites [55], we also investigated whether K13 might be present near these signaling hubs (**Fig 4G** and **S6F Fig**). For these studies, untreated Cam3.II^R539T^ or Cam3.II^WT^ trophozoites were incubated with MitoTracker prior to formaldehyde fixation and co-staining with anti-BiP and anti-K13 antibodies. Interestingly, we frequently observed an overlap of all three labels in both mutant and WT K13 parasites, as indicated in the white dotted outlines (**Fig 4G** and **S6F Fig**).

We next examined whether atovaquone (ATQ), an inhibitor of the *Plasmodium* mitochondrial cytochrome bc1 complex, might affect K13 co-localization with the mitochondria. These studies assayed Cam3.II^R539T^ and Cam3.II^WT^ rings treated for 4h with 60 nM DHA and/or ATQ tested at 100 nM or 1,200 nM concentrations. 60 nM DHA produced no change in PCC values for K13 and MitoTracker compared to DMSO (**Fig 4H and 4I**). 100 nM or 1,200 nM ATQ also generated no change in PCC values between Cam3.II^R539T^ and Cam3.II^WT^ parasites. In contrast, PCC values increased significantly in Cam3.II^R539T^ parasites exposed to a combination of 60 nM DHA and 100 or 1,200 nM ATQ (**Fig 4H and 4I**). This result was observed to a lesser extent in Cam3.II^WT^ parasites but only at the higher ATQ concentration. Elevated ring-stage survival was observed for Cam3.II^R539T^ but not Cam3.II^WT^ parasites following a 6h exposure to 60 nM DHA ± 100 nM ATQ (**S6G Fig**).

### DHA leads to an oxidizing effect in the mitochondria

To further investigate a possible role for parasite mitochondria in the response to DHA, potentially as sensors of DHA-induced oxidative stress, we tested whether DHA treatment results in mitochondrial oxidation. For these experiments, we used the ratiometric redox-sensor hGrx1-roGFP2, consisting of human glutaredoxin 1 fused to an oxidation-reduction sensitive GFP and an N-terminal leader sequence that targets this reporter protein to parasite mitochondria. Assays used *P*. *falciparum* 3D7 parasites episomally transfected with pARL1a-Mito-hGrx1-roGFP2 (referred to herein as 3D7^WT^ Mito-hGrx1-roGFP2) [56] for confocal live-cell imaging. For these studies, we tested DHA, ATQ, and the β-hematin binding drug chloroquine (CQ). Results were compared to mock-treated control parasites. DIA and DTT were included as separate treatments to achieve complete oxidation and reduction, respectively. Results showed that young trophozoites exposed to DHA for 4h underwent a very high degree of mitochondrial oxidation, more than twice the levels observed with ATQ or CQ with similar exposure times and drug concentrations. Increased mitochondrial oxidation with DHA was also observed after 24h. Mitochondrial oxidation was not observed after 30 minutes of parasite exposure to DHA, ATQ or CQ (**Fig 4J**).

## Discussion

With the rise of ART-resistant *P*. *falciparum* parasites in Southeast Asia [11,12], the identification of K13 as the molecular marker of ART resistance represented a key breakthrough in tracking their spread and examining the underlying biological basis of resistance [18]. Here, we have explored the biological role of K13 in DHA- and vehicle-treated asexual blood stage parasites with the use of K13-specific mAbs and recombinant lines expressing epitope-tagged WT or mutant K13. These tools were used in immunofluorescence and IEM-based co-localization studies, and in co-IP experiments to identify putative K13-associated proteins. Our findings suggest an association between K13 and the ER, as well as a role for K13 in vesicular trafficking processes, including cytostomes that transport endocytosed host hemoglobin. We also provide evidence of an association between K13 and mitochondrial proteins and their resident organelle post DHA treatment.

Our IFA results localized K13 to punctate foci that increase in number throughout the parasite IDC. K13 appears to segregate with daughter merozoites, suggesting that the protein is ready to function within very young ring-stage parasites. This observation is consistent with K13-mediated ART resistance occurring in early rings, despite the peak of K13 protein expression occurring in mid trophozoites [17,31,57,58]. Using IEM, we partially co-localized K13 to vesicles in close proximity to the perinuclear ER, the DV and the plasma membrane. We also obtained evidence of K13 localizing near cytostomes, sites where the plasma membrane invaginates to deliver endocytosed hemoglobin to the parasite DV. These results extend prior observations of K13 associating with the ER or vesicular structures as well as sites of cytostomal formation, as defined using GFP-tagged endogenous K13 or polyclonal K13-specific antibodies [25,27,28,35]. Evidence of K13 localizing to cytostomes was also obtained recently using correlative light and electron microscopy [27], a highly-specialized technique that was unavailable for our study. K13 localization patterns were consistent between isogenic WT and mutant Cam3.II lines, as assessed using both K13-specific mAbs and our 3HA- or GFP-tagged NF54^WT^attB lines, indicating that K13 mutations did not affect subcellular localization.

Of note, the NF54^WT^attB-3HA-K13^C580Y^ line, which expresses an integrated mutant K13 allele expressed *in trans*, did not show elevated RSA_0-3h_ survival, suggesting that the endogenous WT isoform is dominant and that K13 polymorphisms might be loss-of-function mutations. RSA data also showed that gene-edited NF54^C580Y^attB-GFP-K13^C580Y^ parasites harboring an integrated second K13 C580Y allele (that results in resistance) reverted to a nearly sensitive phenotype (**Fig 1F**). These findings are consistent with data recently published using episomally transformed K13 mutant parasites [27].

We also observed less labeling of K13 R539T relative to the WT form both by Western blot and by microscopic quantification of K13-positive foci (**S1E and S2C Figs**), consistent with quantitative proteomic analyses showing a ~2-fold decrease in K13 protein abundance in Cam3.II^R539T^ rings as compared to isogenic WT rings [59]. These data suggest that the R539T mutation in this Cam3.II background might reduce K13 protein levels and that this might constitute one causal aspect of mutant K13-mediated ART resistance. Increased overall expression of mutant K13 via co-expression of the endogenous protein and second mutant copy *in trans* thus presumably ablates resistance by compensating for a loss of function in the endogenous locus (**Fig 1F**). In broad agreement with these results, a recent study using K13 conditional knock-sideways parasites showed that mislocalization of K13 can lead to resistance [28]. Further studies will be required to assess whether K13 mutations can differ in their impact on protein stability and activity, how this varies between strains, and to what extent these effects correlate with ART resistance.

Our co-IP experiments resulted in an array of putative K13-associated proteins (**Table 2** and **S2 Table**), suggesting that K13 may interact with multiple proteins across several core pathways including vesicular trafficking, redox regulation and unexpectedly, mitochondrial metabolism and physiology, as discussed below. Our results suggested that few if any interactions were specific to either the WT or mutant K13 isoforms. Many of our candidate K13-associated proteins were also observed in a recent study that used GFP-Trap beads to affinity purify GFP-K13 followed by LC/MS-MS [53]. These proteins included S-adenosylmethionine synthetase (the most abundant protein in our dataset), elongation factor 2, and plasmepsin II. Our list, however, is distinct from the proteins identified in the recent study by Birnbaum et al. [27] that used a quantitative dimerization-induced bio-ID approach (DiQ-BioID) in which GFP-tagged K13 forms a complex with RFP-tagged biotin ligase using dimerization domains regulated by the addition of rapalog. That system enabled biotinylation of K13-proximal proteins, which were then affinity-purified on a streptavidin Sepharose column prior to mass spectrometry and protein identification. One of the proteins identified using this approach, Eps15, was also shown to identify K13 in a reciprocal DiQ-BioID experiment. These proteins were localized to a clathrin-independent AP-2 adaptor complex-labeled compartment involved in hemoglobin endocytosis. Of note, K13 colocalized with AP-2μ, although this protein was not identified by affinity purification of K13 in either the Birnbaum study or our own. A separate study also did not identify K13 upon affinity purification of HA-tagged AP-2μ protein [60]. We cannot yet explain the discrepancy between the data we obtained by co-IP with our K13 mAb and that generated using the DiQ-BioID method, although we note that DiQ-BioID will preferentially identify proteins in the same compartment as K13 rather than proteins physically bound to K13. Our list may have preferentially identified the latter. We also note differences in the protein detergent-based extraction protocols. Further work is clearly required to resolve these differences.

**Fig 5:**
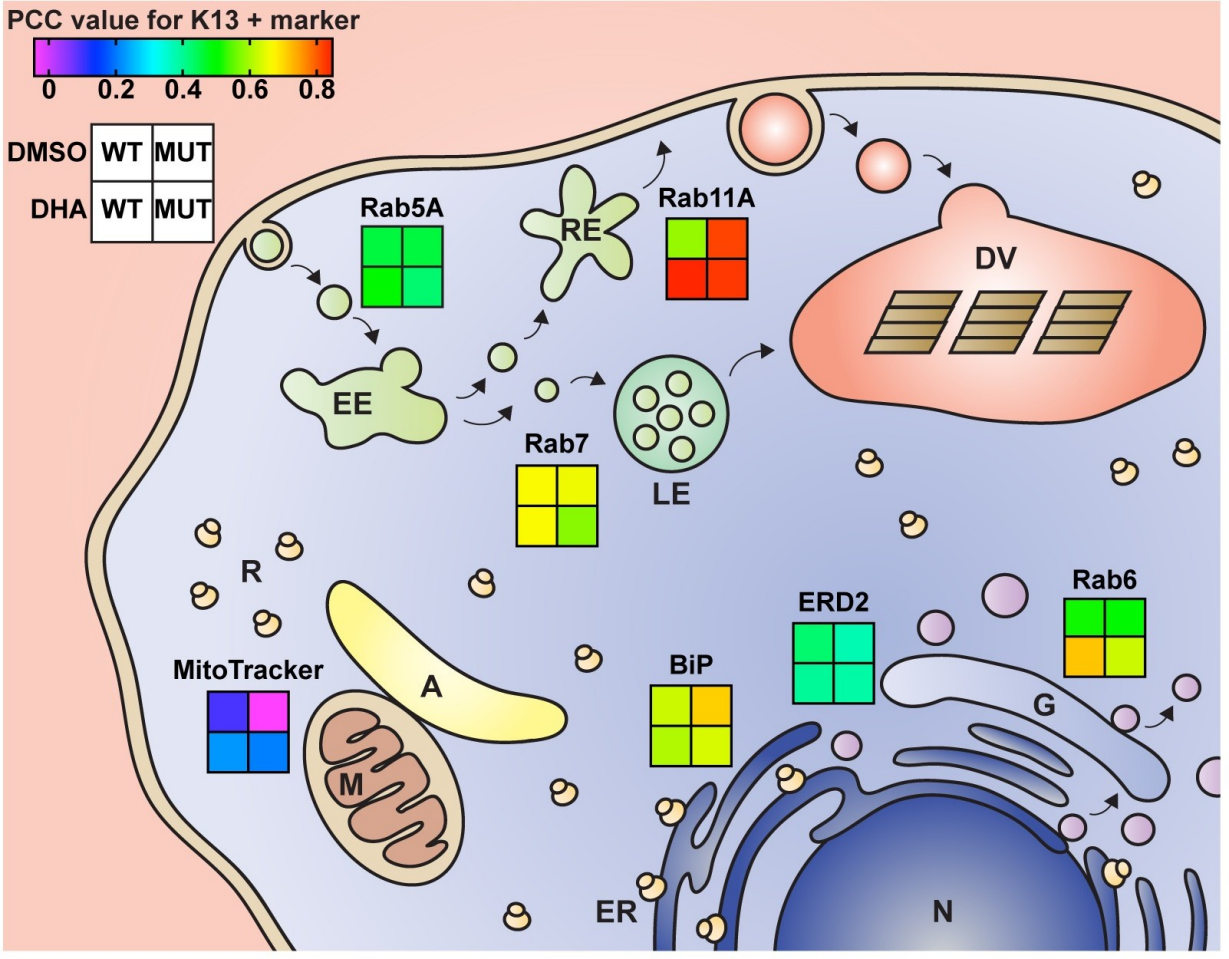
Summary of PCC values for K13 and selected markers upon DHA or DMSO treatment. Schematic showing the subcellular localization of markers used for IFA co-localization studies with K13. The gradient squares illustrate the median PCC values for the spatial association between a given marker and WT or mutant K13. PCC values are shown for DHA- or DMSO-treated parasites and were calculated immediately (0h) post treatment. The Cam3.II^R539T^ and Cam3.II^WT^ lines were employed for all assays, with the exception of those testing the association between K13 and Rab6 that employed the Dd2^R539T^ Rab6-GFP and Dd2^WT^ Rab6-GFP lines. PCC values are also presented in **Figs 2–4**, **S5 Table** and **S6 Table**. A, apicoplast; DHA, dihydroartemisinin; DMSO, dimethyl sulfoxide; DV, digestive vacuole; EE, early endosome; ER, endoplasmic reticulum; G, Golgi apparatus; LE, late endosome; M, mitochondria; MUT, mutant; N, nucleus; PCC, Pearson correlation coefficient; R, ribosome; RE, recycling endosome; WT, wild-type.

Our list of potential interacting partners for K13, based on co-IP and co-localization data, includes multiple Rab GTPases **(Fig 5)**. These included Rabs associated in other eukaryotes with early (Rab5A, 5B, 5C), late (Rab7) or recycling endosomes (Rab 11A, 11B) or the trans-Golgi (Rab6). These and other observed Rabs (e.g. 1A, 1B and 18B) help regulate intracellular cargo trafficking via their association with effector proteins [61,62]. In the case of Rab11A, the consistently high PCC co-localization values with K13 in Cam3.II^R539T^ parasites (**Fig 5**) suggest the possibility of increased export and recycling functions in mutant parasites, which may help eliminate damaged and aggregated proteins during the post-drug recovery phase.

DHA treatment differentially impacted certain correlation values obtained for K13 WT and mutant parasites, as evidenced with Rab5A, B and C. For these, we observed significantly higher PCC values for K13 WT parasites post DHA treatment as compared with K13 R539T mutant parasites. Rab5 proteins have been implicated in hemoglobin import processes, suggesting that the differential associations might impact hemoglobin uptake [63–66]. These findings recall the recent report of reduced heme and heme-DHA adducts in K13 mutant parasites [29,30]. Birnbaum *et al*. also recently showed reduced endocytosis of host cytoplasm, which consists mainly of hemoglobin, in ring-stage parasites expressing mutant K13 (compared to an isogenic WT control) or WT K13 parasites in which this protein was conditionally knocked sideways to cause loss of function [27]. The same study also identified several proteins (including K13, Eps15, UBP1 and AP-2μ) required for hemoglobin endocytosis, of which only K13 was required for rings. This conditional knock sideways system was also recently used by Yang *et al*. to show reduced hemoglobin processing in ring-stage parasites with mislocalized K13 [28]. Stalling of hemoglobin import could have two positive outcomes: less availability of Fe^2+^-heme to activate ART [27,28], and fewer hemoglobin-derived peptides that could trigger a starvation response and entry into a temporary dormant state [67]. Both effects could enable K13 mutant ring stages to survive ART exposure [27,28,68–71].

In mammalian cells, several Rab effector proteins can regulate phosphoinositide metabolism. One of these effectors, a partner of Rab5 and Rab7, is the heterodimeric phosphatidylinositol-3-kinase (PI3K) Vps34/Vps15 complex, which catalyzes the phosphorylation of phosphatidylinositol to phosphatidylinositol-3-phosphate (PI3P) [72–75]. A prior report associated ART resistance with elevated PI3P levels, mediated by an interaction between K13 and PI3K. The rise in PI3P levels in mutant parasites was attributed to reduced binding of K13 to PI3K, resulting in attenuated ubiquitin-mediated degradation of the kinase [34]. PI3K itself was not observed in our co-IP experiments. Nonetheless, there may be a link between PI3P levels and the association we observed between K13 and Rab5 and Rab7, as Rab GTPase activation contributes to the stimulation of PI3K enzymatic activity, leading to localized synthesis of PI3P and regulation of endocytic trafficking [72–75].

The co-localization we observe between the ER chaperone BiP and K13, notably the R539T variant, supports a link between K13 and protein homeostasis and damage response pathways (**Fig 3E**). IEM studies also frequently detected K13 close to the perinuclear parasite ER. These results suggest that mutant K13 might act in part by enhancing ER-associated stress responses to damaged proteins [31,53]. Although BiP co-immunoprecipitated with K13 in all samples across all experiments, it was excluded from our list of putative K13-interacting partners (**Table 2** and **S2 Table**) due to its abundant presence in the negative control samples. Nonetheless, we observed several other ER proteins including PDI-14, Sec61, SEY1 and p97 in our co-IP data, corroborating the IFA and IEM data that partially localized K13 to the ER. We note that interactions with ER-lumenal proteins may arise after detergent-mediated cell lysis, given that K13 lacks a known signal sequence to access the ER lumen.

The ER is not the sole organelle responsible for sensing and combatting cellular stress. Mitochondria likewise regulate an array of cellular functions including ATP production, intracellular calcium buffering, redox homeostasis, and apoptosis [76,77]. There is increasing evidence that the unfolded protein response, generally viewed as a signaling pathway to overcome proteotoxic ER stress, also regulates mitochondrial proteostasis and function [78]. Communication between these two organelles is achieved via specialized mitochondria-associated membrane contact sites (MAMs), where Ca^2+^ transfer, lipid synthesis and autophagosome assembly take place. In times of stress, Ca^2+^ flow into the mitochondria can augment mitochondrial respiratory chain activity, increasing energy resources to mount an adaptive stress response [78]. Our co-IP data revealed multiple putative K13-interacting mitochondrial proteins whose functions span oxidative stress responses, the electron transport chain, and mitochondrial protein synthesis (**Table 2** and **S2 Table**). Without DHA, an association between K13 and the mitochondria was essentially nonexistent, but PCC values for K13 and MitoTracker increased significantly post DHA treatment, especially for the K13 R539T mutant. This putative association was not tested in the Birnbaum *et al*. study, which did not examine whether treatment with an ART derivative would alter their set of K13-interacting candidates [27]. We caution that our co-IP data associating K13 with mitochondrial proteins might be adversely affected by detergent-based extraction conditions that could lead to false associations with this organelle. Nonetheless, we observed partial colocalization of K13 with MitoTracker-stained mitochondria upon DHA treatment, and note a recent study with *Toxoplasma gondii* parasites that provided evidence of ART targeting the mitochondria, where it affected membrane potential and organelle morphology [79].

The association between K13 and the mitochondria that we observed recalls earlier evidence of ART accumulating in this organelle, as well as in the DV, causing mitochondrial swelling as early as 2h post drug exposure [80]. The mode of action of ART and other endoperoxides has also been linked to the rapid depolarization of the mitochondrial membrane potential, with surviving cells maintaining mitochondrial polarization and activity despite widespread cellular damage [81,82]. Mitochondrial membrane depolarization was attributed to the formation of reactive oxygen species, most likely originating from iron-mediated bioactivation of the ART endoperoxide bridge. Using a genetically-encoded, mitochondria-targeted GFP-fusion redox probe, we found substantial oxidation following 4h or 24h of DHA treatment, which greatly exceeded any oxidative effect of the cytochrome bc1 inhibitor ATQ or the hemozoin inhibitor CQ. These results agree with earlier studies that also showed an impact of ART derivatives on redox potential in the parasite cytosol [56,83,84]. Further studies are merited to elucidate the role of the mitochondria in both DHA action and K13-mediated resistance. One possibility is that this organelle might act as an initial sensor of ART action, with mutant K13 altering mitochondrial functionalities in ways that have downstream impacts across an array of pathways, including reversible entry into quiescence during ART exposure or subsequent recovery of resistant parasites. Further studies into the connection between K13 function and the mitochondria could substantially advance our understanding of parasite physiology and its capacity to counter ART-mediated proteotoxic and oxidative stress.

## Materials and methods

### Production of monoclonal antibodies to K13

Antibodies were raised against K13 by injecting mice intraperitoneally with two types of immunogens: recombinant BTB plus propeller domain (~40 kDa) or recombinant propeller domain alone (~32 kDa) (**Fig 1A**). Immunogens were kindly provided by Dr. Raymond Hui (University of Toronto). Mice were immunized five times at three-week intervals. Sera were collected 9–10 days after the last immunization, and titers of anti-K13 IgG were measured by ELISA using His-tagged versions of the recombinant K13 proteins bound to Ni^2+^-coated ELISA plates. Mice with the highest antibody titers were selected for anti-K13 hybridoma populations, which were generated via polyethylene glycol (PEG)-induced fusion of the MEP-2S fusion partner cell line with murine splenic B cells. Hybridoma cell lines were maintained in RPMI-1640 medium supplemented with 10% FBS, L-glutamine, non-essential amino acids, sodium pyruvate and vitamins. Stable clones were selected in the presence of hypoxanthine-aminopterine-thymidine (HAT) medium. Hybridoma populations producing K13-specific antibodies, as determined by ELISA, were expanded and cloned to assure the monoclonal nature of antibodies. Clones selected via ELISA were further screened for their K13 specificity by IFAs with the NF54^WT^attB-GFP-K13^WT^ line. Clones were also tested by Western blot against the immunogens. Purified clonal mAbs were generated from K13-positive hybridomas.

### Plasmid construction

For GFP-K13 expression studies, the *K13* WT coding sequence (PlasmoDB ID PF3D7_1343700) was amplified from genomic DNA using p3947 and p3948 (**S7 Table**) and cloned into a pDC2-based expression system downstream of GFP using the BglII and XhoI restriction sites [42]. The *K13* 5’ untranslated region (UTR) was amplified as a 2000 bp fragment using p4376 and p4377 (**S7 Table**) and cloned into the ApaI and AvrII restriction sites. We then fused the full-length *K13* coding sequence to an N-terminal GFP tag and placed this sequence under the regulatory control of the endogenous promoter. This plasmid was named pDC-2000-GFP-K13^WT^-bsd-attP. For 3HA-K13 expression studies, the *K13* C580Y coding sequence was used instead, and the *P*. *berghei* *ef1*α promotor (PBANKA_1133400) was used as a 5’ regulatory element. The N-terminal GFP tag was replaced with an N-terminal 3HA tag at the AvrII and BglII sites. The 3HA sequence was synthetically engineered with the corresponding restriction sites in a pUC57-Amp vector (Genewiz). The resulting plasmid was named pDC2-EF1α-3HA-K13^C580Y^-bsd-attP. In both plasmids *hsp86* 3’UTR was used as a terminator sequence for the *K13* expression cassette and a *bsd* (blasticidin S-deaminase) cassette was used as a selectable marker [85].

To episomally express the *GFP-Rab6* transgene we amplified the *Rab6* coding sequence (PlasmoDB ID PF3D7_1144900) from genomic DNA using poML214 and poML204 (**S7 Table**) and cloned this fragment into the pDC2-based expression system downstream of GFP using the BglII and XhoI restriction sites [42]. Using p1144 and p1263 (**S7 Table**), about 1.3 kb of the *Sec12* (PlasmoDB ID PF3D7_1116400) 5’ UTR was amplified as the promoter and cloned into the ApaI and AvrII restriction sites. The *hsp86* 3’UTR served as a terminator sequence for the expression cassette and human *dhfr* (dihydrofolate reductase) was used as the selectable marker. The plasmid was named pDC2-sec12-GFP-PfRab6-hDHFR.

### Parasite culture and transfection

*P*. *falciparum* asexual blood-stage parasites were cultured in human erythrocytes (3% hematocrit) and RPMI-1640 medium supplemented with 2 mM L-glutamine, 50 mg/L hypoxanthine, 25 mM HEPES, 0.225% NaHCO_3_, 10 mg/L gentamycin and 0.5% w/v Albumax II (Invitrogen). Parasites were maintained at 37°C in 5% O_2_, 5% CO_2_, and 90% N_2_. Cultures were stained with Giemsa, and monitored by blood smears fixed in methanol and viewed by light microscopy.

NF54^WT^attB-GFP-K13^WT^ and NF54^WT^attB-3HA-K13^C580Y^ parasite lines were generated by attB×attP crossover-mediated integration of either the pDC-2000-GFP-K13^WT^-bsd-attP or the pDC2-EF1α-3HA-K13^C580Y^-bsd-attP plasmid into the *cg6* locus in the NF54^WT^attB parasite line [86] (**S1 Fig**). Crossover events were mediated by Bxb1 serine integrase, which was expressed on the pINT plasmid that contains the neomycin selectable marker and that was co-electroporated with either pDC2 plasmid. The NF54^C580Y^attB-GFP-K13^C580Y^ line was generated using a *K13*-specific CRISPR/Cas9 system that enabled us to edit both the endogenous and transgene copies of *K13* in the NF54^WT^attB-GFP-K13^WT^ line (Stokes *et al*., manuscript in preparation). This system was also used to generate the NF54^WT^attB-3HA-K13^WT^ line by CRISPR/Cas9-editing the *K13* C580Y transgene locus in the NF54^WT^attB-3HA-K13^C580Y^ line. Dd2^WT^ or Dd2^R539T^ GFP-Rab6 parasite lines were generated via transfection of the pDC2-sec12-GFP-PfRab6-hDHFR plasmid and episomal selection using 2.5 nM WR99210 (Jacobus Pharmaceuticals, Princeton, NJ).

Transfections were performed by electroporating ring-stage parasites at 5–10% parasitemia with 50 μg of purified circular plasmid DNA in resuspended in Cytomix [87]. Transfected parasites were maintained under 2.5 μg/mL blasticidin (Thermo Fisher) or 2.5 nM WR99210 drug pressure to select for maintenance of the pDC2-based *bsd* or h*dhfr* plasmids respectively, and 125 μg/mL G418 (Fisher) to select for pINT. Blasticidin or WR99210 pressure was maintained until parasites were detected microscopically, whereas G148 pressure was applied for only the first six days post electroporation. Parasite cultures were monitored by microscopy for up to six weeks post electroporation. To test for successful integration of the attP plasmids, trophozoite-infected erythrocytes were harvested and saponin-lysed, and genomic DNA was isolated using QIAamp DNA Blood Mini kit (Qiagen). PCR-based screening for integration is shown in **S1 Fig**, with primers listed in **S7 Table**. Integrated parasites were cloned via limiting dilution, and flow cytometry was used to screen for positive wells after 17–20 days. Parasites were stained with 1×SYBR Green (Thermo Fisher) and 100 nM MitoTracker Deep Red (Invitrogen) and detected using an Accuri C6 flow cytometer (Becton Dickinson; [88]). Expression of the tagged proteins in the clonal lines was verified via Western blot and IFA with anti-GFP or anti-HA primary antibodies, as described below.

### Western blotting

Parasite lysates for Western blotting were prepared on ice. Infected erythrocytes were washed twice in cold 1× phosphate-buffered saline (PBS), and parasites were isolated from RBCs by treatment with 0.05% saponin in PBS. Released parasites were resuspended in cold lysis buffer (0.15 M NH_4_Cl, 1 mM NaHCO_3_, 0.1 mM Na_2_EDTA, pH 7.4) supplemented with 1× protease inhibitors (Halt Protease Inhibitors Cocktail, Thermo Fisher) and incubated on ice for 30 min. RBC membranes were lysed with 0.1% Triton X100 in PBS supplemented with protease inhibitors for 10 min, with frequent vortexing and trituration. Samples were centrifuged at 14,000 rpm for 10 min at 4°C to pellet cellular debris. Supernatants were collected and protein concentrations were determined using the DC protein assay kit (Bio-Rad). Laemmli Sample Buffer (Bio-Rad) was added to lysates and samples were denatured at 90°C for 10 min. Proteins were electrophoresed on 10% Bis-Tris gels (Bio-Rad) and transferred onto a nitrocellulose membrane. Western blots were probed with primary antibodies to K13 or to GFP or 3×HA epitope tags (1:1,000 dilutions for all) and incubated with HRP-conjugated secondary antibodies (1:10,000 dilution). Western blots were revealed using ECL Western Blotting Substrate (Thermo Fisher). For primary antibodies, we used Living Colors full-length anti-GFP polyclonal antiserum (Takara (Clontech)), anti-HA antibodies produced in rabbit (Sigma), rabbit anti-ERD2 antibodies (BEI Resources) and mouse anti-β actin antibodies (clone AC-15, Invitrogen). As secondary antibodies, we used goat anti-rabbit IgG H&L (HRP) (Abcam) or goat anti-mouse IgG H&L (HRP) (Abcam).

### Immunofluorescence assays

Parasites were synchronized with 5% D-sorbitol treatment and harvested either every 12h throughout the 48h life cycle, or were pulsed with DHA (6h, 700 nM) or Brefeldin A (1h, 5 μg/mL) (Sigma Aldrich) and then harvested post treatment. DMSO was used as vehicle control in the case of DHA treatments and EtOH in the case of BFA (**S3 Fig**). DHA-treated parasites were harvested either immediately (0h) post treatment, or 3, 12, or 24h post treatment. BFA-treated parasites were harvested 1h post treatment.

IFAs were performed with cells in suspension. Harvested cells were washed twice in 1×PBS and fixed in 4% v/v formaldehyde (Fisher) supplemented with 0.0075% v/v glutaraldehyde (Sigma) in PBS for 30 min at room temperature. Cell membranes were permeabilized in 0.1% Triton X-100 in PBS for 30 min. Autofluorescence was quenched using 0.1 M glycine in PBS for 15 min. Blocking was performed with 3% w/v bovine serum albumin (BSA) for at least 1h at room temperature, or overnight at 4°C. Cells were incubated with primary antibodies for 90 min at room temperature or overnight at 4°C, with dilutions ranging from 1:50–1:200, followed by incubation with a species-specific fluorophore-conjugated secondary antibody (Alexa Fluor 488-, 594- or 647-conjugated goat anti-mouse, -rabbit or -rat antibody, Thermo Fisher) diluted 1:2,000 to 1:4,000 in 3% BSA and 0.1% Tween in PBS. As primary antibodies, we used rabbit anti-BiP (kindly provided by Min Zhang), rabbit anti-ERD2 (BEI Resources), rabbit anti-Rab5A, -Rab5C, or -Rab11A, rat anti-Rab5B or -Rab7 (kindly provided by Dr. Gordon Langsley), rabbit anti-TRiC (kindly provided by Zbynek Bozdech), rabbit anti-HAD1 (kindly provided by Dr. Audrey Odom John), rabbit anti-GFP ((Takara (Clontech)), or rabbit anti-HA (Sigma). MitoTracker Red CMXRos (Thermo Fisher) was used to stain mitochondria.

Thin blood smears of stained RBCs were prepared on microscope slides and mounted with cover slips using Prolong Diamond Antifade Mountant with DAPI (Thermo Fisher). Slides were imaged using a Nikon Eclipse Ti-E wide-field microscope equipped with a sCMOS camera (Andor) and a Plan-apochromate oil immersion objective with 100× magnification (1.4 numerical aperture). A minimum of 15 Z-stacks (0.2 μm step size) were taken for each parasitized RBC. NIS-Elements imaging software (Version 5.02, Nikon) was used to control the microscope and camera as well as to deconvolve images and perform 3D reconstructions. Deconvolution was performed using 25 iterations of the Richardson-Lucy algorithm for each image. Quantitative co-localization analysis of the deconvolved Z-stacks was performed using the GA3 pipeline (General analysis Pipeline 3; NIS-Elements software; developed in collaboration with Nikon). ImageJ (Fiji version 2.0.0-rc-68/1.52h) was used to crop images, adjust brightness and intensity, overlay channels and prepare montages. For super resolution imaging, we used either a Nikon N-SIM S Super Resolution Microscope or a W1-Yokogawa Spinning Disk Confocal with a CSU-W1 SoRa Unit. For 3D image analysis, we used Imaris x64 version 6.7 (Bitplane).

### Immunoelectron microscopy

Trophozoites were magnetically sorted from uninfected RBCs and ring-stage parasites via MACS LD separation columns (Miltenyi Biotech). Parasites were collected by centrifugation and fixed for 1h at 4°C in 4% paraformaldehyde (Polysciences Inc.) in 100 mM PIPES with 0.5 mM MgCl_2_ (pH 7.2). Samples were embedded in 10% gelatin and infiltrated overnight with 2.3 M sucrose and 20% polyvinyl pyrrolidone in PIPES/MgCl_2_ at 4°C. Samples were trimmed, frozen in liquid nitrogen, and sectioned with a Leica Ultracut UCT7 cryo-ultramicrotome (Leica Microsystems Inc.). 50 nm sections were blocked with 5% fetal bovine serum and 5% normal goat serum for 30 min, and subsequently incubated with primary antibodies for 1h at room temperature (anti-PDI (1D3) mouse diluted 1:50, anti-GFP rabbit 1:200, anti-GFP mouse 1:200, anti-HA rabbit 1:50–1:250, anti-BiP rabbit 1:100, anti-Rab5A rabbit 1:50, anti-Rab5B rat 1:50, or anti-Rab7 rabbit 1:50; Rab11A antibodies could not be used as they failed to give signals under our conditions). Species-specific colloidal-gold conjugated secondary antibodies (6 nm, 12 nm or 18 nm particles; Jackson ImmunoResearch) were added at a 1:30 dilution for 1h at room temperature. Sections were stained with 0.3% uranyl acetate and 2% methyl cellulose, and viewed on a JEOL 1200 EX transmission electron microscope (JEOL USA Inc.) equipped with an AMT 8-megapixel digital camera and AMT Image Capture Engine V602 software (Advanced Microscopy Techniques). All labeling experiments were conducted in parallel with controls omitting the primary antibodies. These controls were consistently negative at the concentration of colloidal gold-conjugated secondary antibodies used in these studies.

### Co-immunoprecipitation (Co-IP) studies

Co-IP studies were performed using the Pierce Direct IP kit (Thermo Fisher). Briefly, parasites were extracted from infected erythrocytes as described above, resuspended in Pierce IP Lysis Buffer supplemented with 1× Halt Protease and Phosphatase Inhibitor Cocktail and 25U Pierce Universal Nuclease, and lysed on ice for 10 minutes with frequent vortexing. Samples were centrifuged at 14,000 rpm for 10 min at 4°C to pellet cellular debris. Supernatants were collected and protein concentrations were determined using the DC protein assay kit (Bio-Rad). IPs were performed used 500 μg of lysate per test sample. A mix of K13 E3 and D9 mAbs (2.5 μg each per test sample) was used for IP. Antibody coupling to IP columns, IP, and elution steps were performed according to Pierce instructions. Eluates were analyzed by liquid chromatography-tandem mass spectrometry (LC-MS/MS) to identify immunoprecipitated proteins.

### Ring-stage survival assays (RSA_0-3h_)

RSA_0-3h_ assays were carried out as previously described [17], with minor modifications. In brief, parasite cultures were synchronized 1–2 times using 5% sorbitol (Fisher). Synchronous schizonts were incubated in RPMI-1640 containing 15 units/mL sodium heparin (Fisher) for 15 min at 37°C to disrupt agglutinated erythrocytes, concentrated over a gradient of 75% Percoll (Fisher), washed once in RPMI-1640, and incubated for 3h with fresh RBCs to allow time for merozoite invasion. Cultures were subjected again to sorbitol treatment to eliminate remaining schizonts. 0–3h post-invasion rings were adjusted to 1% parasitemia and 2% hematocrit and exposed to 700 nM DHA or 0.1% DMSO (vehicle control) for 6h. Alternatively, early rings were exposed to 4h to 60 nM, 100 nM or 1200 nM ATQ, or combinations thereof. These concentrations were selected based on separate studies from our lab showing synergy between DHA and ATQ against Cam3.II^R539T^ parasites at these concentrations. Cells were washed to remove drug and returned to standard culture conditions for an additional 66h. Parasite growth in each well was assessed using flow cytometry. Parasites were stained with 1x SYBR Green and 100 nM MitoTracker Deep Red (Thermo Fisher), and parasitemias were measured on a BD Accuri C6 Plus Flow Cytometer with a HyperCyt attachment sampling 60,000–100,000 events per well. After 72h, cultures generally expanded to 3–5% parasitemia in DMSO-treated controls. Percent survival of DHA-treated parasites was calculated relative to the corresponding DMSO-treated control.

### Confocal live-cell imaging

*P*. *falciparum* 3D7 parasites were episomally transfected with pARL1a-Mito-hGrx1-roGFP2, which expresses an oxidation-reduction sensitive GFP fused at its N-terminus to human glutaredoxin 1 (**Table 1**). This fusion protein is targeted to the mitochondria using the citrate synthase leader sequence, as described previously [56]. Transfected parasites were used to test the oxidizing effects of antimalarials on the parasite mitochondria. For 30 min exposure experiments we incubated trophozoites with 100 μM DHA, ATQ, or CQ. For mid-term 4h incubations we exposed young trophozoites to 5 μM DHA, ATQ, or CQ. Long-time 24h exposures began with young ring-stage parasites, which were exposed to 50 nM DHA, ATQ, or CQ.

Following drug exposure, parasites were blocked with 2 mM N-ethylmaleimide (NEM) for 15 min. Trophozoite-stage parasites were magnetically enriched and eluted in pre-warmed Ringer’s solution and seeded on poly-lysine coated μ-slides VI (Ibidi, Martinsried, Germany). Live-cell imaging was performed on a Leica confocal system TCS SP5 inverted microscope equipped with an HCX PL APO 63.0 x 1.30 GLYC 37°C UV objective and a 37°C temperature chamber, as previously described [83]. Smart gain was set to 222.0 V, smart offset was 12.3% and argon laser power was set to 20%. To calibrate the microscope, we used parasites whose redox state was either fully reduced with 10 mM DTT or fully oxidized with 1 mM DIA. Data were analyzed using Leica LAS AF software. The degree of mitochondrial oxidation (OxD) was calculated as follows:

$$\mathrm{OxD}=\frac{R‑R_{\mathrm{red}}}{\frac{I_{488\mathrm{ox}}}{I_{488\mathrm{red}}}\left(R_{\mathrm{ox}}‑R\right)+(R‑R_{\mathrm{red}})}$$

R represents the ratio of the fluorescence intensity measured at 405 nm and 488 nm ($R=\frac{405\;\mathrm{nm}}{488\;\mathrm{nm}}$); R_red_ and R_ox_ are the ratios of the fluorescence intensity of fully reduced or fully oxidized parasites, respectively; I_488ox_ is the fluorescence intensity at 488 nm for fully oxidized parasites; and I_488red_ is the fluorescence intensity at 488 nm for fully reduced parasites [89]. Graphs were plotted using GraphPad Prism version 8.

### Ethics statement

Human RBCs used in this study were purchased from the Interstate Blood Bank (Memphis, TN) as whole blood from anonymized donors. Approval to use this material for *P*. *falciparum in vitro* culture was granted by the Columbia University Medical Center Institutional Review Board, which has classified this work as not being human subjects research. The use of mice in this study was described in protocol AC-AAM8301, which was reviewed and approved by the Columbia University Institutional Animal Care and Use Committee. Our animal use and care protocol adheres to the NIH Guidelines for Pain and Distress in Laboratory Animals.

## Supporting information

S1 Fig Generation of NF54^WT^attB-GFP-K13^WT^ and NF54^WT^attB-3HA-K13^C580Y^ parasites. (A) Schematic of GFP-K13^WT^ or 3HA-K13^C580Y^ gene sequence integration into NF54^WT^ parasites containing an attB site in the *cg6* locus [90]. The two plasmids used for co-transfection are represented at the top. pINT codes for the integrase expression unit (Int) and a neomycin resistance cassette (Neo). pDC-2000-GFP-K13^WT^-bsd-attP contains an N-terminal GFP-K13^WT^ fusion protein under the control of the endogenous *K13* promoter (*k13* 5’UTR), and a blasticidin S-deaminase (BSD) resistance cassette adjacent to the attP coding site. pDC-EF1α-3HA-K13^C580Y^-bsd-attP contains an N-terminal 3HA-K13^C580Y^ fusion protein under the control of the *pbef1α* promoter, and a BSD resistance cassette. Integrase-mediated recombination between the attP and attB sequences resulted in integration of the full-length pDC2-based plasmids, yielding the NF54^WT^attB**-**GFP-K13^WT^ and NF54^WT^attB-3HA-K13^C580Y^ transgenic parasite lines. **(B)** Primer combinations and expected amplicon sizes used for PCR-based integration screening. Primer positions are indicated with arrows in **(A)** and primer sequences are listed in **S7 Table**. **(C)** PCR analysis of the two transgenic lines using the primer sets listed in **(B)**. **(D)** Western blots of parasite extracts probed with the anti-K13 mAb E9. This antibody recognizes full-length K13 (~85 kDa) and lower molecular weight bands. We attribute the latter to N-terminal degradation products, based on our observation of very high co-localization values between K13 mAbs and antibodies to either GFP or 3HA in K13 transgenic lines, as well as the finding that antibodies to GFP or 3HA both recognized fusion proteins consistent with a K13 mass of ~85 kDa (as seen in **Fig 1A**). **(E)** Representative Western blot analysis of synchronized 0-6h ring-stage parasites from the K13- isogenic lines Cam3.II^WT^, Cam3.II^C580Y^ and Cam3.II^R539T^, probed with K13 mAb E9 and mouse monoclonal anti-β actin. The right panel shows ImageJ-generated quantification of K13 C580Y or K13 R539T protein compared to K13 WT protein, with all proteins normalized to the β-actin loading control. These data yielded relative mean ± SEM expression levels of 76 ± 3% and 66 ± 4% for Cam3.II^C580Y^ and Cam3.II^R539T^ relative to the WT control, corresponding to mean K13 protein percent reductions of 24% and 34% for these two mutant proteins respectively.
(PDF)

S2 Fig Additional super resolution imaging of **(A)** Cam3.II^WT^ and **(B)** Cam3.II^R539T^ trophozoites, labeled with antibodies to K13 and the cytosolic marker HAD1. Images were acquired using a W1-Yokogawa Spinning Disk Confocal microscope equipped with a CSU-W1 SoRa Unit. **(C)** Quantification of antibody-labeled K13 foci in Cam3.II^WT^ and Cam3.II^R539T^ trophozoites, yielding an estimated 48% reduction in K13 R539T protein compared to the K13 WT levels.
(PDF)

S3 Fig Schematic of the protocol used for synchronizing and treating parasites for immunofluorescence co-localization studies. DHA, dihydroartemisinin; DMSO, dimethyl sulfoxide; MACS, magnetic-activated cell sorting.
(PDF)

S4 Fig K13 partially co-localizes with Rab GTPases and Sec24a. **(A)** Representative IFA images showing DMSO-treated Cam3.II^WT^ ring-stage parasites co-stained with anti-K13 mAb E3 and antibodies to Rab5A, Rab5B, or Rab5C (top, middle and bottom panels, respectively). Samples were collected immediately post treatment. Scale bars: 2 μm. **(B)** Fluorescence microscopy/DIC overlay and 3D volume reconstruction showing the spatial association between K13 and Rab5A in Cam3.II^WT^ parasites sampled 12h post DMSO mock treatment. Scale bars are indicated. **(C)** Representative IFA images showing GFP-Rab6-expressing parasites co-stained with K13 mAb E3. Assays were conducted with Dd2^WT^ (top) and Dd2^R539T^ (bottom) ring-stage parasites episomally expressing GFP-Rab6, and samples were collected immediately post DMSO treatment. Scale bars: 2 μm. **(D)** Representative IFA images showing DMSO-treated Cam3.II^WT^ ring-stage parasites co-stained with anti-K13 mAb E3 and antibodies to Rab7 (top) or Rab11A (bottom). Samples were collected immediately post treatment. Scale bars: 2 μm. **(E)** Fluorescence microscopy/DIC overlay and 3D volume reconstruction showing the spatial association between K13 and Rab11A in Cam3.II^WT^ parasites sampled 12h post DMSO treatment. **(F)** Representative IEM images of NF54^WT^attB-GFP-K13^WT^ (left) or NF54^WT^attB-3HA-K13^C580Y^ (right) trophozoites stained with anti-GFP or anti-HA antibodies, and either co-stained with antibodies to Rab5A (top), or Rab5B (bottom left), or triply labeled with anti-Rab5B and anti-PDI antibodies (bottom right). Arrows highlight locations of interest. ER, endoplasmic reticulum; Hz, Hemozoin; M, mitochondria; N, nucleus. Scale bars: 100 nm. **(G)** PCC values for the spatial association between K13 and Sec24a immediately post DHA pulse (6h, 700 nM) or DMSO mock treatment. Assays were conducted on Dd2^WT^ ring-stage parasites episomally expressing Sec24a-GFP. Parasites were stained with anti-GFP and the K13 mAb E3. Right panels show representative 3D volume reconstructions of DMSO-treated or DHA-pulsed Sec24a-GFP expressing parasites. PCC values were calculated and statistics performed as in **Fig 2**. Scale bars: 1 μm. **(H)** Representative IFA images showing Dd2^WT^ Sec24a-GFP-expressing parasites co-stained with K13 mAb E3 and anti-GFP. Samples were collected immediately post DMSO mock treatment. Scale bars: 2 μm. Several DIC images as well as montages showing the individual color channels complement the 3D volume view of parasites shown in **Fig 2**.
(PDF)

S5 Fig K13 exhibits extensive co-localization with the parasite ER. **(A)** Fluorescence microscopy/DIC overlay and 3D volume reconstructions of deconvolved Z-stacks showing the spatial association between K13 and BiP in Cam3.II^WT^ (top) and Cam3.II^R539T^ (bottom) trophozoites (untreated). Parasites were co-stained with the K13 E3 mAb and anti-BiP antibodies. Scale bars: 2 μm. **(B)** Representative IEM images of NF54^WT^attB-GFP-K13^WT^ trophozoites co-stained with anti-GFP and anti-BiP antibodies. Arrows highlight locations of interest. Hz, hemozoin; N, nucleus. Scale bars: 100 nm. **(C)** PCC values for the spatial association of K13 and BiP in Cam3.II^R539T^ and Cam3.II^WT^ ring-stage parasites treated and analyzed as in **Fig 3B–3E**. **(D)** Representative IFA images showing Cam3.II^WT^ ring-stage parasites co-stained with anti-K13 E3 and either anti-BiP (left) or anti-ERD2 (right) antibodies. Parasites were sampled immediately post DHA pulse (6h, 700 nM) or DMSO mock treatment. Scale bars: 2 μm.
(PDF)

S6 Fig K13 localizes to mitochondria-associated membranes. **(A)** PCC values for the association of K13 with parasite mitochondria in NF54^WT^attB-GFP-K13^WT^ ring-stage parasites co-stained with MitoTracker Deep Red and anti-GFP. Samples were collected either 0h or 12h post DHA pulse (6h, 700 nM). DMSO was used as a vehicle control. PCC values were calculated and statistics performed as in **Fig 2**. **(B-E)** PCC values for the association of **(B)** ERD2, **(C)** TRiC, **(D)** Rab5A, or **(E)** Rab11A with parasite mitochondria in Cam3.II^R539T^ and Cam3.II^WT^ ring-stage parasites. Samples were collected 0h post DHA pulse (6h, 700nM). Parasites were co-stained with MitoTracker Deep Red and marker-specific antibodies. **(F)** Additional representative 3D volume reconstructions of untreated late (left) Cam3.II^R539T^ and (right) Cam3.II^WT^ trophozoites triply stained with MitoTracker, anti-BIP (ER, green) and anti-K13 E3 (purple). White dotted outlines indicate spatial overlap between the three labels. Scale bars: 1 μm. **(G)** Percent survival for Cam3.II^R539T^ and Cam3.II^WT^ 0–3 hpi rings treated for 4h with DHA and/or ATQ at the concentrations indicated (in nM). Data show mean ± SEM for three independent experiments performed in duplicate.
(PDF)

S1 Video 3D rotations of a Cam3.II^WT^ late trophozoite labeled with anti-K13 (green) and anti-HAD1 (red) antibodies.
(MP4)

S1 Table Co-immunoprecipitation experimental details. (PDF)

S2 Table Putative K13-interacting protein partners identified by co-immunoprecipitation and LC/MS-MS (relaxed criteria). (PDF)

S3 Table PANTHER overrepresentation test for biological processes. (PDF)

S4 Table PANTHER overrepresentation test for cellular components. (PDF)

S5 Table Pearson correlation coefficient values for IFA studies. (PDF)

S6 Table Pearson correlation coefficient values for MitoTracker Deep Red imaging studies. (PDF)

S7 Table Oligonucleotides used in this study. (PDF)
