# Supplementary material for: Insights into the intracellular localization, protein associations and artemisinin resistance properties of Plasmodium falciparum K13
Source: PLoS Pathog. 2020 Apr 20;16(4):e1008482. doi: 10.1371/journal.ppat.1008482 (PMC7192513; doi:10.1371/journal.ppat.1008482)

Figure S1

A

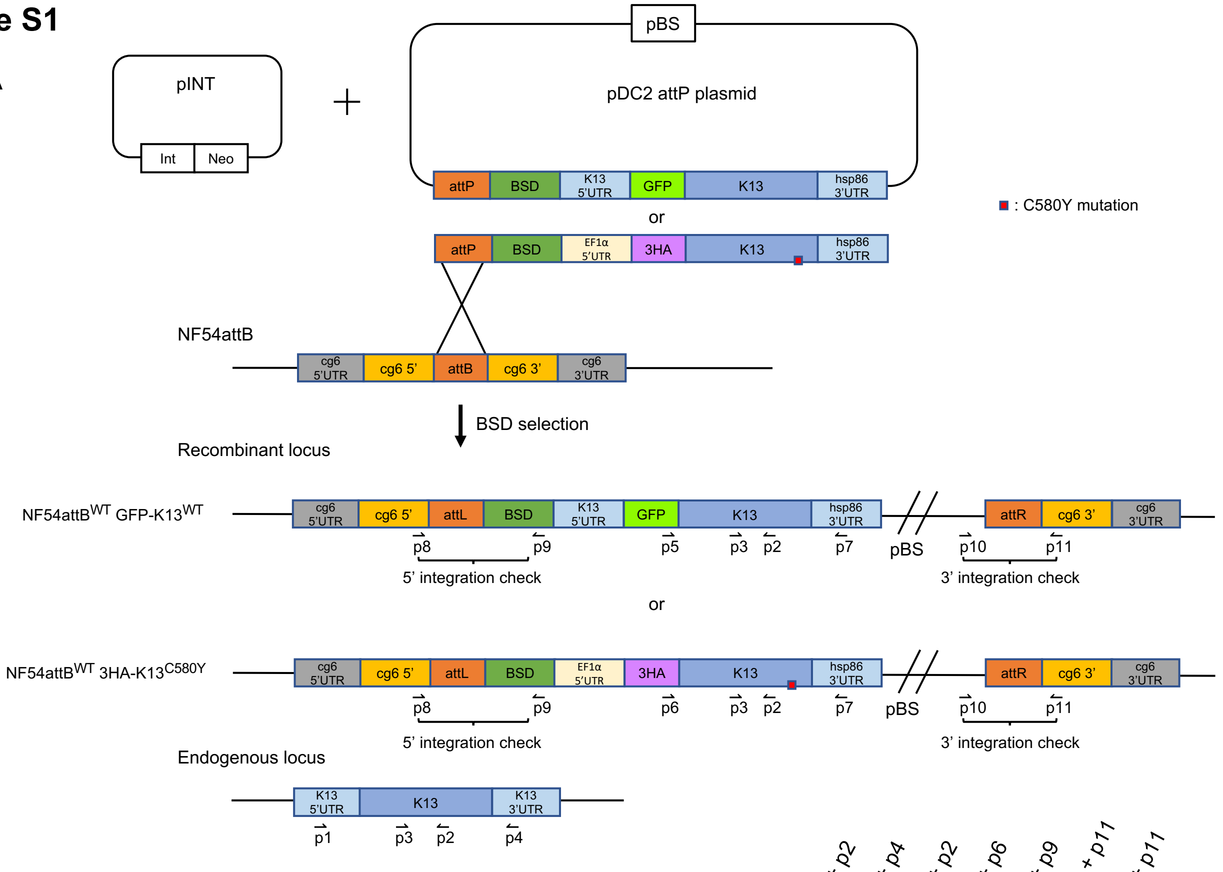

B

locus	primer pair	size
endogenous 5'	p1 + p2	2.6 kb
endogenous 3'	p3 + p4	2.0 kb
GFP recombinant 5'	p5 + p2	1.2 kb
3HA recombinant 5'	p6 + p2	1.5 kb
recombinant 3'	p3 + p7	1.2 kb
attB integration 5'	p8 + p9	1.7 kb
attB integration 3'	p10 + p11	532 bp
NF54attB cg6 locus	p8 + p11	200 bp

C

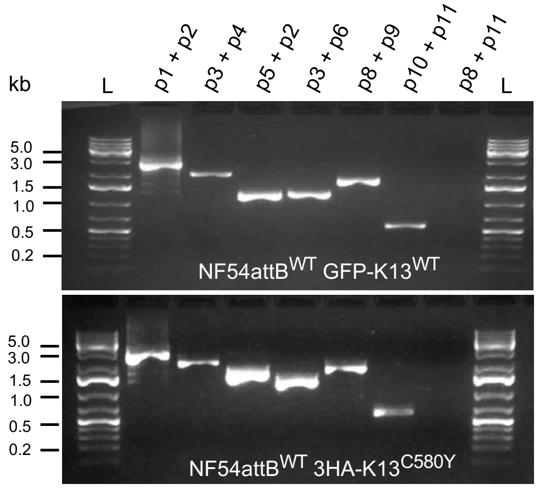

D

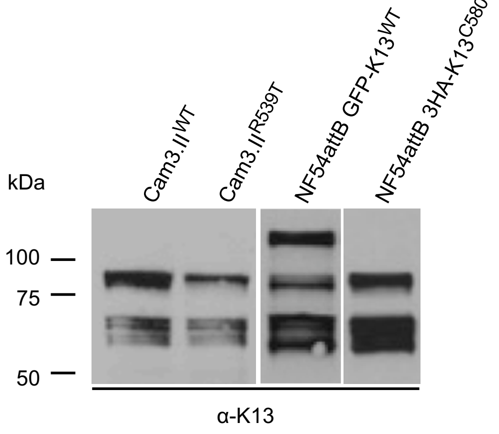

E

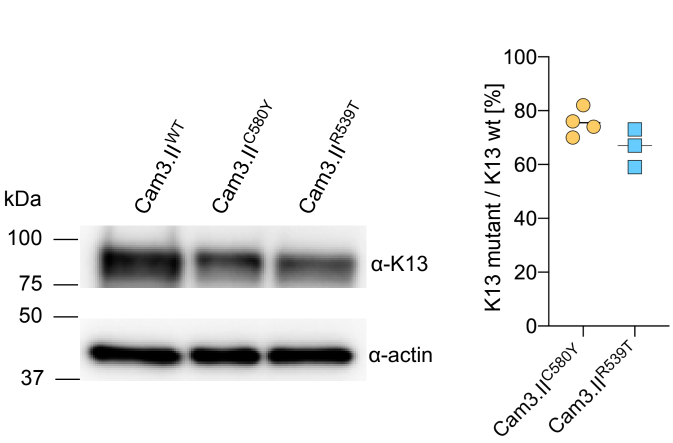

Supplement: S1 Fig — (A) Schematic of GFP-K13WT or 3HA-K13C580Y gene sequence integration into NF54WT parasites containing an attB site in the cg6 locus [90]. The two plasmids used for co-transfection are represented at the top. pINT codes for the integrase expression unit (Int) and a neomycin resistance cassette (Neo). pDC-2000-GFP-K13WT-bsd-attP contains an N-terminal GFP-K13WT fusion protein under the control of the endogenous K13 promoter (k13 5’UTR), and a blasticidin S-deaminase (BSD) resistance cassette adjacent to the attP coding site. pDC-EF1α-3HA-K13C580Y-bsd-attP contains an N-terminal 3HA-K13C580Y fusion protein under the control of the pbef1α promoter, and a BSD resistance cassette. Integrase-mediated recombination between the attP and attB sequences resulted in integration of the full-length pDC2-based plasmids, yielding the NF54WTattB-GFP-K13WT and NF54WTattB-3HA-K13C580Y transgenic parasite lines. (B) Primer combinations and expected amplicon sizes used for PCR-based integration screening. Primer positions are indicated with arrows in (A) and primer sequences are listed in S7 Table. (C) PCR analysis of the two transgenic lines using the primer sets listed in (B). (D) Western blots of parasite extracts probed with the anti-K13 mAb E9. This antibody recognizes full-length K13 (~85 kDa) and lower molecular weight bands. We attribute the latter to N-terminal degradation products, based on our observation of very high co-localization values between K13 mAbs and antibodies to either GFP or 3HA in K13 transgenic lines, as well as the finding that antibodies to GFP or 3HA both recognized fusion proteins consistent with a K13 mass of ~85 kDa (as seen in Fig 1A). (E) Representative Western blot analysis of synchronized 0-6h ring-stage parasites from the K13- isogenic lines Cam3.IIWT, Cam3.IIC580Y and Cam3.IIR539T, probed with K13 mAb E9 and mouse monoclonal anti-β actin. The right panel shows ImageJ-generated quantification of K13 C580Y or K13 R539T protein compared to K13 WT prot [file ppat.1008482.s001.pdf]
