# Supplementary figures and images for: Insights into the intracellular localization, protein associations and artemisinin resistance properties of Plasmodium falciparum K13
Source: PLoS Pathog. 2020 Apr 20;16(4):e1008482. doi: 10.1371/journal.ppat.1008482 (PMC7192513; doi:10.1371/journal.ppat.1008482)

**Figure S2**

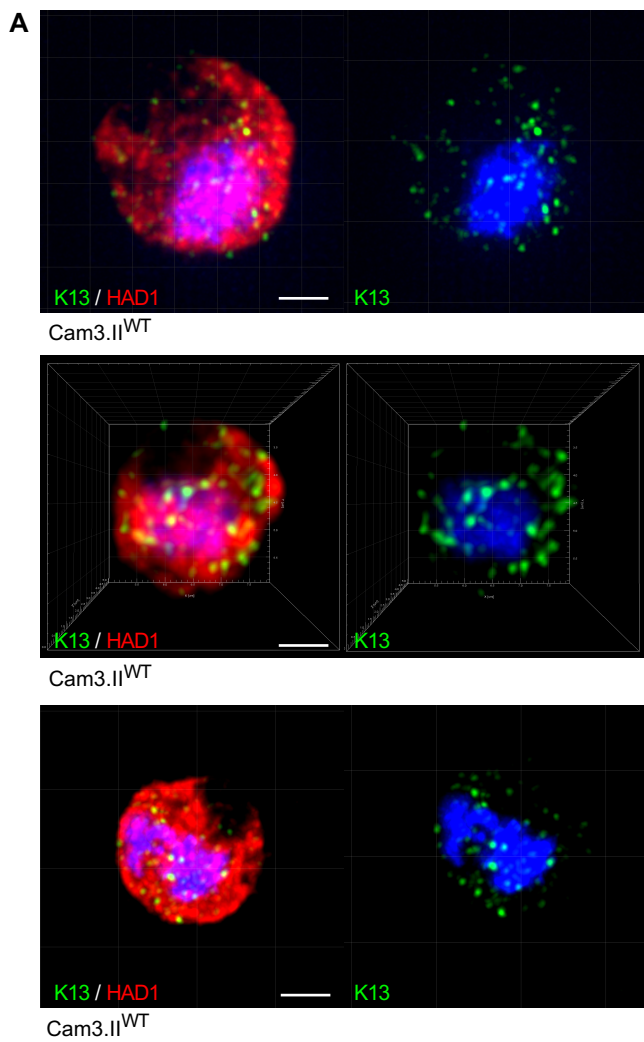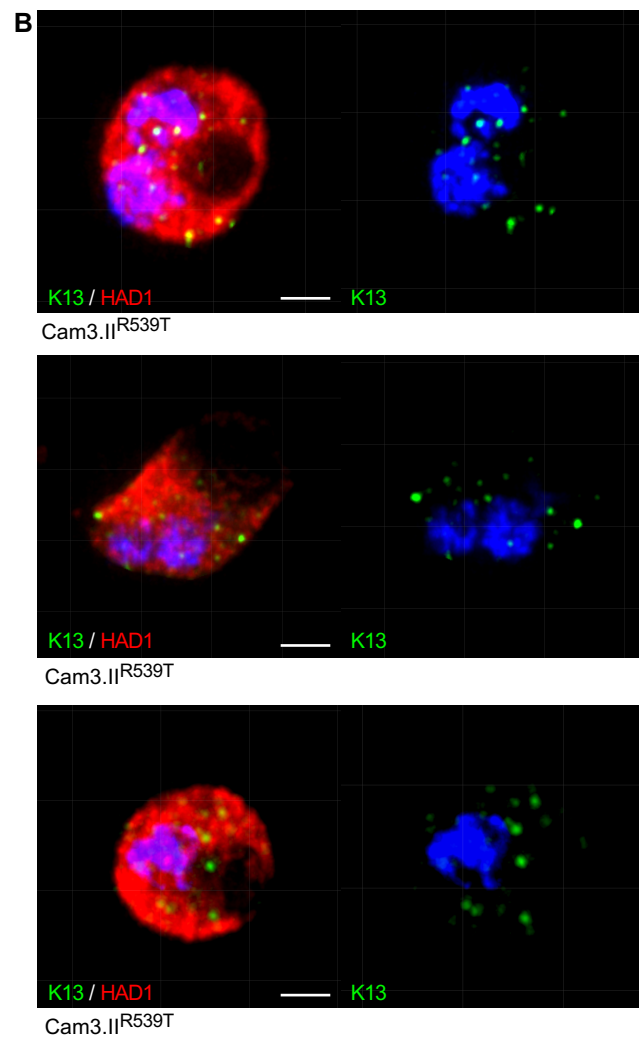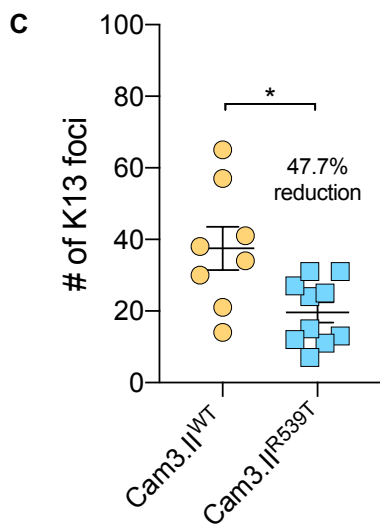

Supplement: S2 Fig — Additional super resolution imaging of (A) Cam3.IIWT and (B) Cam3.IIR539T trophozoites, labeled with antibodies to K13 and the cytosolic marker HAD1. Images were acquired using a W1-Yokogawa Spinning Disk Confocal microscope equipped with a CSU-W1 SoRa Unit. (C) Quantification of antibody-labeled K13 foci in Cam3.IIWT and Cam3.IIR539T trophozoites, yielding an estimated 48% reduction in K13 R539T protein compared to the K13 WT levels. (PDF) [file ppat.1008482.s002.pdf]

**Figure S3**

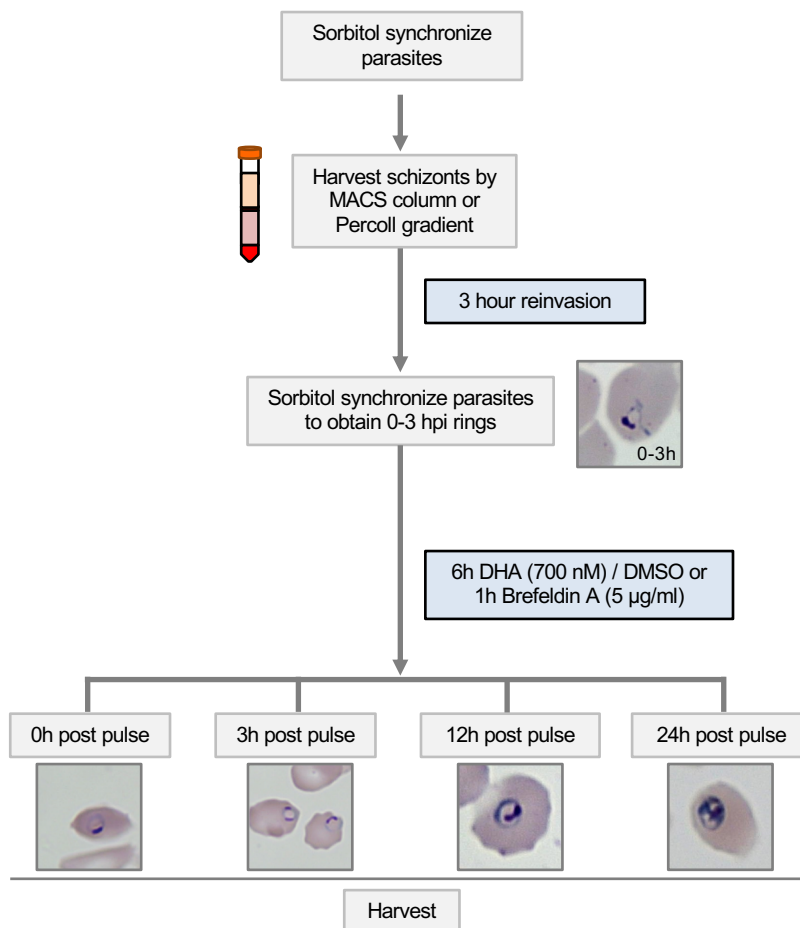

Supplement: S3 Fig — DHA, dihydroartemisinin; DMSO, dimethyl sulfoxide; MACS, magnetic-activated cell sorting. (PDF) [file ppat.1008482.s003.pdf]

**Figure S5****A**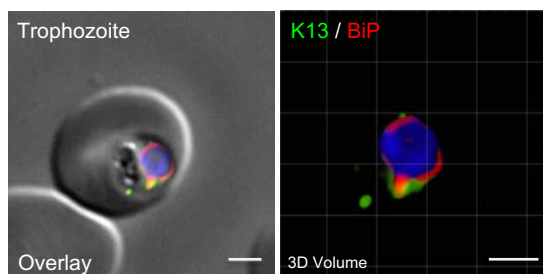Cam3.II<sup>WT</sup>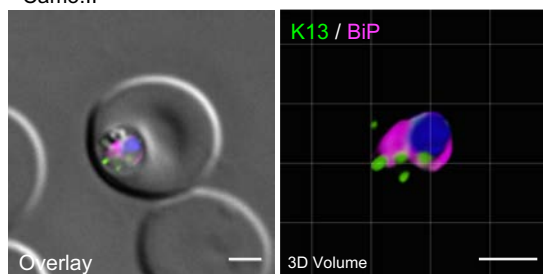Cam3.II<sup>R539T</sup>**B**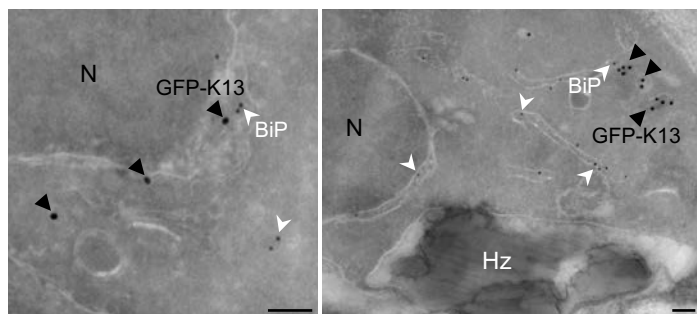NF54attB<sup>WT</sup> GFP-K13<sup>WT</sup>**C**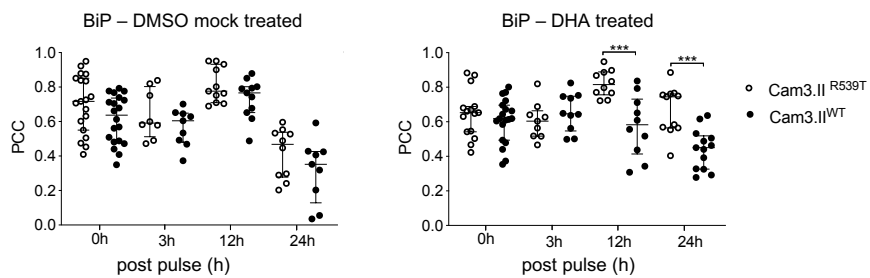**D**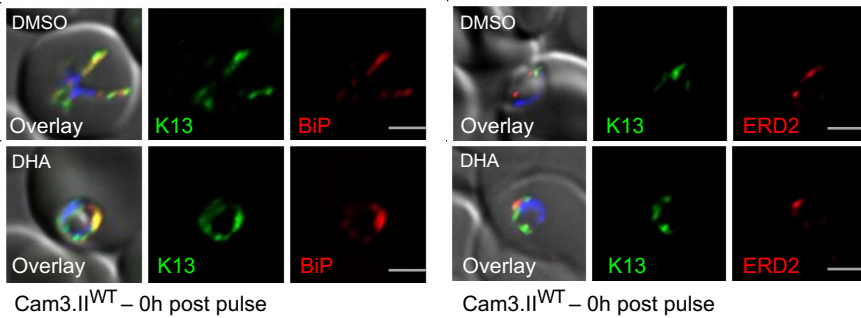

Supplement: S5 Fig — (A) Fluorescence microscopy/DIC overlay and 3D volume reconstructions of deconvolved Z-stacks showing the spatial association between K13 and BiP in Cam3.IIWT (top) and Cam3.IIR539T (bottom) trophozoites (untreated). Parasites were co-stained with the K13 E3 mAb and anti-BiP antibodies. Scale bars: 2 μm. (B) Representative IEM images of NF54WTattB-GFP-K13WT trophozoites co-stained with anti-GFP and anti-BiP antibodies. Arrows highlight locations of interest. Hz, hemozoin; N, nucleus. Scale bars: 100 nm. (C) PCC values for the spatial association of K13 and BiP in Cam3.IIR539T and Cam3.IIWT ring-stage parasites treated and analyzed as in Fig 3B–3E. (D) Representative IFA images showing Cam3.IIWT ring-stage parasites co-stained with anti-K13 E3 and either anti-BiP (left) or anti-ERD2 (right) antibodies. Parasites were sampled immediately post DHA pulse (6h, 700 nM) or DMSO mock treatment. Scale bars: 2 μm. (PDF) [file ppat.1008482.s005.pdf]

**Figure S6**

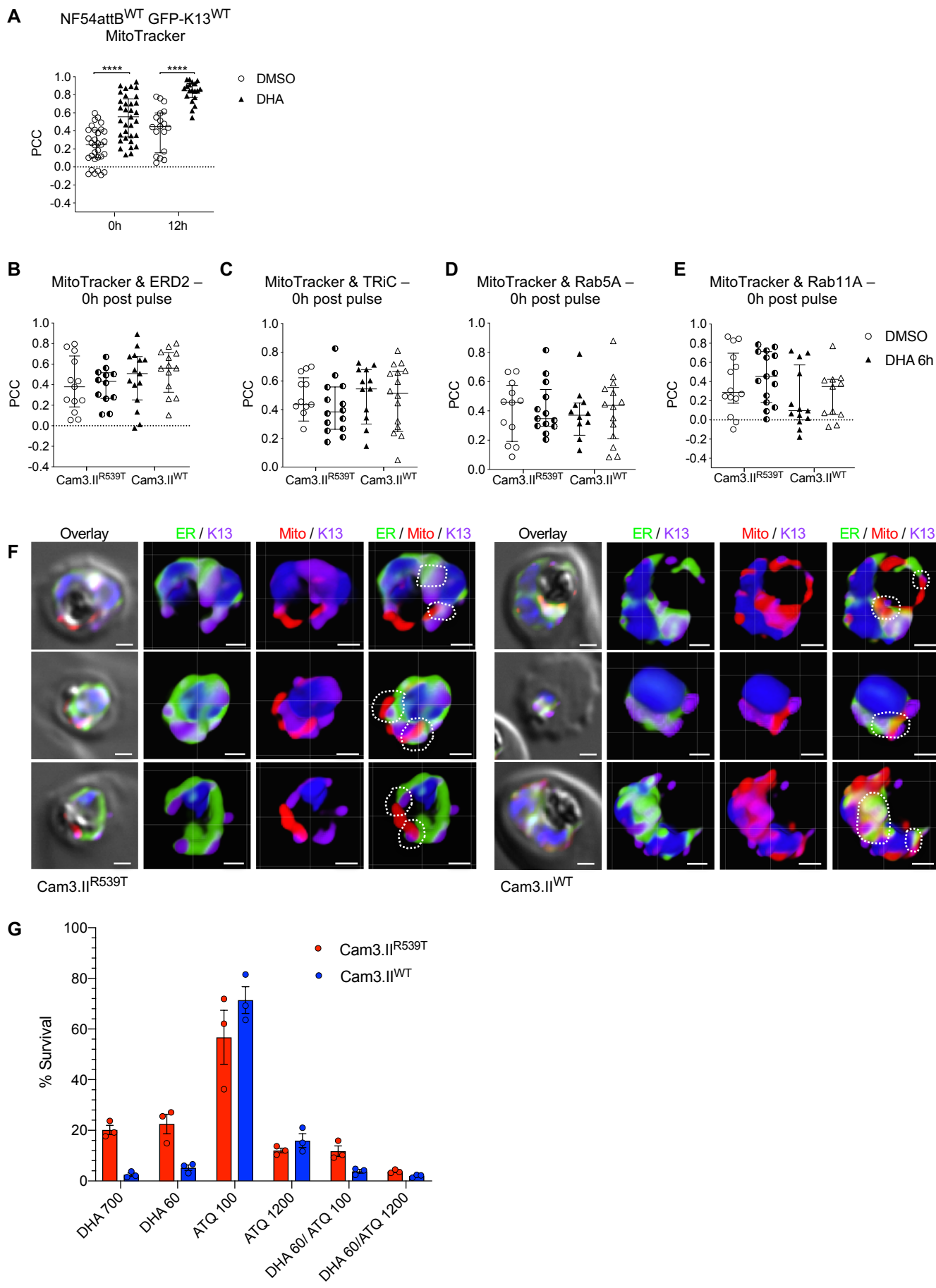

Supplement: S6 Fig — (A) PCC values for the association of K13 with parasite mitochondria in NF54WTattB-GFP-K13WT ring-stage parasites co-stained with MitoTracker Deep Red and anti-GFP. Samples were collected either 0h or 12h post DHA pulse (6h, 700 nM). DMSO was used as a vehicle control. PCC values were calculated and statistics performed as in Fig 2. (B-E) PCC values for the association of (B) ERD2, (C) TRiC, (D) Rab5A, or (E) Rab11A with parasite mitochondria in Cam3.IIR539T and Cam3.IIWT ring-stage parasites. Samples were collected 0h post DHA pulse (6h, 700nM). Parasites were co-stained with MitoTracker Deep Red and marker-specific antibodies. (F) Additional representative 3D volume reconstructions of untreated late (left) Cam3.IIR539T and (right) Cam3.IIWT trophozoites triply stained with MitoTracker, anti-BIP (ER, green) and anti-K13 E3 (purple). White dotted outlines indicate spatial overlap between the three labels. Scale bars: 1 μm. (G) Percent survival for Cam3.IIR539T and Cam3.IIWT 0–3 hpi rings treated for 4h with DHA and/or ATQ at the concentrations indicated (in nM). Data show mean ± SEM for three independent experiments performed in duplicate. (PDF) [file ppat.1008482.s006.pdf]
