# Supplementary material for: Insights into the intracellular localization, protein associations and artemisinin resistance properties of Plasmodium falciparum K13
Source: PLoS Pathog. 2020 Apr 20;16(4):e1008482. doi: 10.1371/journal.ppat.1008482 (PMC7192513; doi:10.1371/journal.ppat.1008482)
Supplement: S4 Fig — (A) Representative IFA images showing DMSO-treated Cam3.IIWT ring-stage parasites co-stained with anti-K13 mAb E3 and antibodies to Rab5A, Rab5B, or Rab5C (top, middle and bottom panels, respectively). Samples were collected immediately post treatment. Scale bars: 2 μm. (B) Fluorescence microscopy/DIC overlay and 3D volume reconstruction showing the spatial association between K13 and Rab5A in Cam3.IIWT parasites sampled 12h post DMSO mock treatment. Scale bars are indicated. (C) Representative IFA images showing GFP-Rab6-expressing parasites co-stained with K13 mAb E3. Assays were conducted with Dd2WT (top) and Dd2R539T (bottom) ring-stage parasites episomally expressing GFP-Rab6, and samples were collected immediately post DMSO treatment. Scale bars: 2 μm. (D) Representative IFA images showing DMSO-treated Cam3.IIWT ring-stage parasites co-stained with anti-K13 mAb E3 and antibodies to Rab7 (top) or Rab11A (bottom). Samples were collected immediately post treatment. Scale bars: 2 μm. (E) Fluorescence microscopy/DIC overlay and 3D volume reconstruction showing the spatial association between K13 and Rab11A in Cam3.IIWT parasites sampled 12h post DMSO treatment. (F) Representative IEM images of NF54WTattB-GFP-K13WT (left) or NF54WTattB-3HA-K13C580Y (right) trophozoites stained with anti-GFP or anti-HA antibodies, and either co-stained with antibodies to Rab5A (top), or Rab5B (bottom left), or triply labeled with anti-Rab5B and anti-PDI antibodies (bottom right). Arrows highlight locations of interest. ER, endoplasmic reticulum; Hz, Hemozoin; M, mitochondria; N, nucleus. Scale bars: 100 nm. (G) PCC values for the spatial association between K13 and Sec24a immediately post DHA pulse (6h, 700 nM) or DMSO mock treatment. Assays were conducted on Dd2WT ring-stage parasites episomally expressing Sec24a-GFP. Parasites were stained with anti-GFP and the K13 mAb E3. Right panels show representative 3D volume reconstructions of DMSO-treated or DHA-pulsed Sec24a-GFP expressing [file ppat.1008482.s004.pdf]

Figure S4

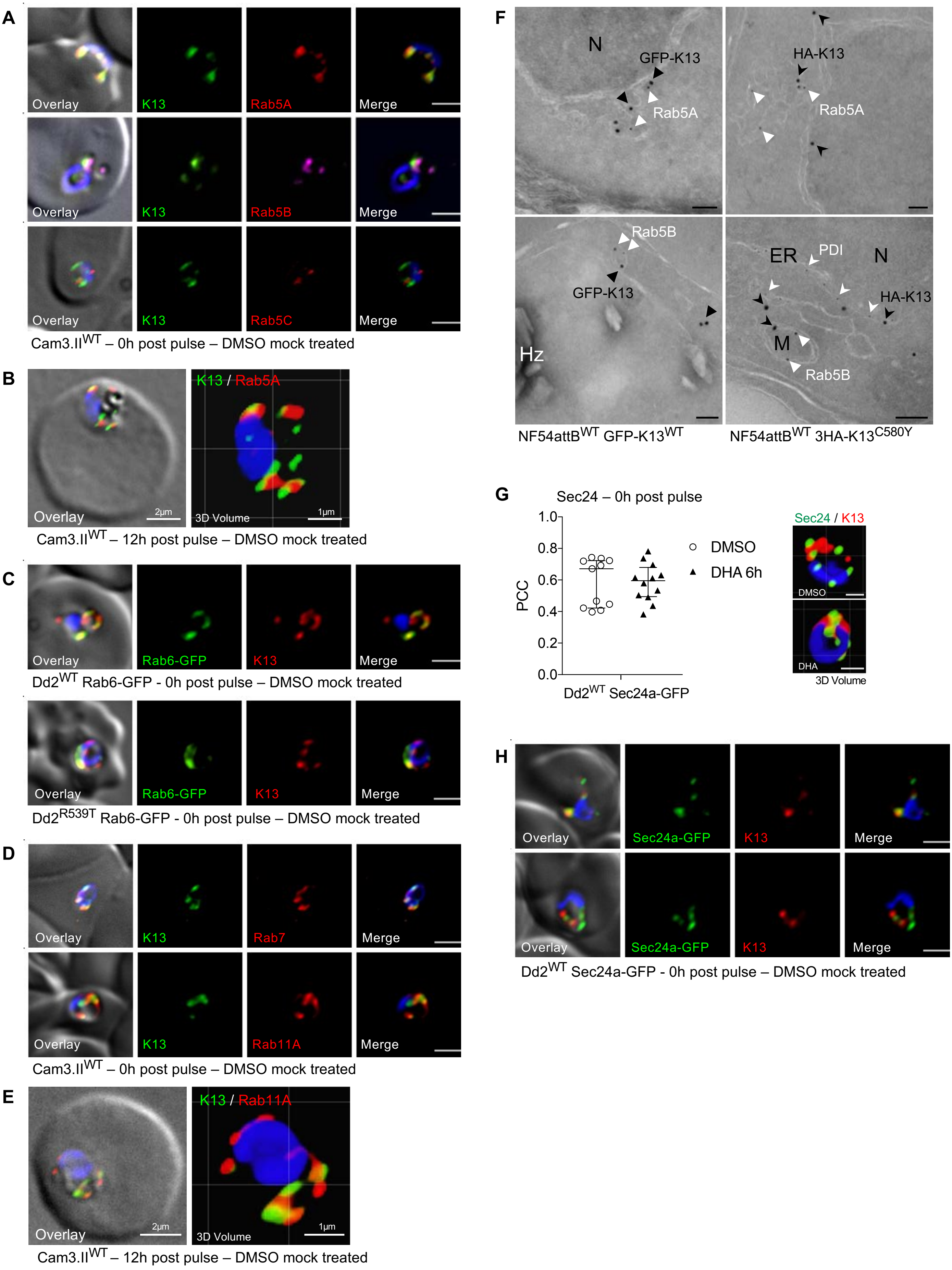
A
Overlay
K13
Rab5A
Merge
Rab5B
Rab5C
Cam3.II^WT – 0h post pulse – DMSO mock treated
B
K13 / Rab5A
2µm
1µm
3D Volume
Cam3.II^WT – 12h post pulse – DMSO mock treated
C
Rab6-GFP
Dd2^WT Rab6-GFP - 0h post pulse – DMSO mock treated
Dd2^R539T Rab6-GFP - 0h post pulse – DMSO mock treated
D
Rab7
Rab11A
Cam3.II^WT – 0h post pulse – DMSO mock treated
E
K13 / Rab11A
Cam3.II^WT – 12h post pulse – DMSO mock treated
F
N
GFP-K13
HA-K13
Hz
ER
PDI
M
NF54attB^WT GFP-K13^WT
NF54attB^WT 3HA-K13^C580Y
G
Sec24 – 0h post pulse
PCC
1.0
0.8
0.6
0.4
0.2
0.0
DMSO
DHA 6h
Dd2^WT Sec24a-GFP
Sec24 / K13
DHA
H
Sec24a-GFP
Dd2^WT Sec24a-GFP - 0h post pulse – DMSO mock treated
