# Supplementary material for: Insights into the intracellular localization, protein associations and artemisinin resistance properties of Plasmodium falciparum K13
Source: PLoS Pathog. 2020 Apr 20;16(4):e1008482. doi: 10.1371/journal.ppat.1008482 (PMC7192513; doi:10.1371/journal.ppat.1008482)
Supplement: S1 Table — (PDF) [file ppat.1008482.s008.pdf]

S1 Table. Co-immunoprecipitation experimental details.

Experiment Number	Parasite lines included	K13 mAbs used	Negative controls included
1	Cam3.II^{WT}, Cam3.II^{C580Y}, Cam3.II^{R539T}	E3, D9	--
2	CamWT	E3, D9	--
3	Cam3.II^{WT}, Cam3.II^{C580Y}, Cam3.II^{R539T}	E3, D9	Control resin, Control IgG[1]
4	CamWT, CamWT^{C580Y}	E3, D9	Control resin
5	Cam3.II^{WT}, Cam3.II^{R539T}	E3, D9	Control resin
6	Cam3.II^{WT}, Cam3.II^{R539T}	E3, D9	Control resin

[1]Control resin refers to Pierce^{TM} Control Agarose Resin (Pierce^{TM} Direct IP Kit) only with no antibody coupled to IP column. Control IgG refers to AminoLink Plus Coupling Resin (PierceTM Direct IP Kit) with an irrelevant mouse-specific IgG coupled to the IP column.
