# Supplementary material for: Insights into the intracellular localization, protein associations and artemisinin resistance properties of Plasmodium falciparum K13
Source: PLoS Pathog. 2020 Apr 20;16(4):e1008482. doi: 10.1371/journal.ppat.1008482 (PMC7192513; doi:10.1371/journal.ppat.1008482)
Supplement: S2 Table — (PDF) [file ppat.1008482.s009.pdf]

S2 Table. Putative K13-interacting protein partners identified by co-immunoprecipitation and LC/MS-MS (relaxed criteria). Page 1 of 2.

PlasmoDB Gene ID	Gene Name	Abbreviation	Cellular component and/or functional features	Mean fold change	Number of experiments present (of 6)[1]	Number of samples present (of 13 total, 6 WT, 7 mutant)[2]
PF3D7_0708400	Heat shock protein 90	HSP90	Oxidative protein folding in the ER, component of chaperone complexes that interact with BiP	298	5	11 (5,6)
PF3D7_1312600	2-oxoisovalerate dehydrogenase subunit alpha, mitochondrial, putative	BCKDHA	Mitochondrial TCA cycle	270	5	8 (3,5)
PF3D7_1408000	Plasmepsin II	PMII	Digestive vacuole hemoglobinase	264	5	11 (5,6)
PF3D7_1324900	L-lactate dehydrogenase	LDH	Glycolysis	173	4	9 (4,5)
PF3D7_0933600	Mitochondrial-processing peptidase subunit beta, putative	MAS1	Mitochondrial protein degradation	166	5	8 (4,4)
PF3D7_1020900	ADP-ribosylation factor	ARF1	Intracellular traffic, Clathrin, COPI	106	5	9 (3,6)
PF3D7_0523100	Mitochondrial-processing peptidase subunit alpha, putative	MAS2	Mitochondrial protein degradation	92	5	8 (4,4)
PF3D7_0207600	Serine repeat antigen 5	SERA5	Parasitophorous vacuole, parasite egress	87	5	9 (5,4)
PF3D7_0935900	Ring-exported protein 1	REX1	Maurer's cleft exported protein	81	4	6 (2,4)
PF3D7_1446200	M17 leucyl aminopeptidase	LAP	Hemoglobin digestion	76	6	10 (5,5)
PF3D7_1345700	Isocitrate dehydrogenase [NADP], mitochondrial	IDH	Mitochondrial antioxidant system / TCA cycle	74	6	13 (6,7)
PF3D7_0904800	Replication protein A1, small fragment	RPA1	DNA replication / repair	70	3	7 (2,5)
PF3D7_1034400	Flavoprotein subunit of succinate dehydrogenase	SDHA	Mitochondrial respiratory chain complex II (ETC)	60	5	10 (5,5)
PF3D7_0303700	Lipoamide acyltransferase component of branched-chain alpha-keto acid dehydrogenase complex	BCKDH-E2	Mitochondrial antioxidant system	59	3	5 (3,2)
PF3D7_0617200	Conserved *Plasmodium* protein, unknown function	--	Brf1p family coiled coil protein homolog, putative	59	3	8 (3,5)
PF3D7_0513800	Ras-related protein Rab1A	RAB1A	ER-Golgi translocation and quality control	53	3	6 (2,4)
PF3D7_0919000	Nucleosome assembly protein	NAP1	Histone exchange during transcription elongation	50	4	7 (2,5)
PF3D7_0629200	DnaJ protein, putative	--	Oxidative protein folding in the ER, component of chaperone complexes that interact with BiP	47	6	13 (6,7)
PF3D7_0217100	ATP synthase F1, alpha subunit	--	Mitochondrion, proton-transporting ATP synthase complex, Ca2+ homeostasis	44	4	7 (3,4)
PF3D7_0619400	Cell division cycle protein 48 homologue, putative	p97	ER-associated protein degradation (ERAD)	41	6	11 (5,6)
PF3D7_1239600	Hydroxyethylthiazole kinase	ThzK	Thiamine (Vitamin B) metabolism / glucose metabolism	40	4	9 (4,5)
PF3D7_1246200	Actin I	ACT1	Actin filaments	38	6	13 (6,7)
PF3D7_1022400	Serine/arginine-rich splicing factor 4	SR4	Regulation of mRNA splicing, RNA steady state levels	36	2	5 (2,3)
PF3D7_1302800	40S ribosomal protein S7, putative	--	Ribosome	36	5	9 (4,5)
PF3D7_0103900	Parasite-infected erythrocyte surface protein	ERGIC-53	Intracellular traffic	36	3	6 (3,3)
PF3D7_1118200	Heat shock protein 90, putative	HSP90	Mitochondrial inner membrane	36	6	12 (5,7)
PF3D7_1218500	Dynamin-like protein, putative	--	Mitochondrial division	33	5	9 (4,5)
PF3D7_1340700	Ras-related protein Rab11B	RAB11B	Intracellular traffic / Exocytosis	33	4	9 (4,5)
PF3D7_0817500	histidine triad nucleotide-binding protein 1	--	Ca2+ homeostasis	32	4	7 (4,3)
PF3D7_1108500	Succinyl-CoA synthetase alpha subunit, putative	--	Mitochondrial antioxidant system / TCA cycle	31	6	11 (5,6)
PF3D7_0935800	Cytoadherence linked asexual protein 9	CLAG9	Invasion molecule	30	5	8 (3,5)
PF3D7_0811600	Conserved Plasmodium protein, unknown function	--	No known or predicted function	28	5	7 (4,3)
PF3D7_0302500	Cytoadherence linked asexual protein 3.1	CLAG3.1	Invasion molecule	27	4	6 (4,2)
PF3D7_1407100	rRNA 2'-O-methyltransferase fibrillarin, putative	NOP1	Nucleolus, small nucleolar ribonucleoprotein complex	27	4	8 (3,5)
PF3D7_1144900	Ras-related protein Rab6	RAB6	Intracellular traffic / trans-Golgi network	27	4	8 (3,5)
PF3D7_1230400	ATP-dependent protease subunit ClpQ	ClpQ	Mitochondrial protease complex, protein export	27	6	12 (6,6)
PF3D7_0306400	FAD-dependent glycerol-3-phosphate dehydrogenase, putative	--	Mitochondrial respiratory chain complex II (ETC)	26	4	9 (4,5)
PF3D7_0310400	Parasite-infected erythrocyte surface protein	PIESP1	Exported protein, host cell plasma membrane	25	3	5 (2,3)
PF3D7_1464700	ATP synthase (C/AC39) subunit, putative	--	Digestive vacuole, vacuolar proton-transporting V-type ATPase, Ca2+ homeostasis	25	4	7 (3,4)
PF3D7_1362200	RuvB-like helicase 3	RUVB3	Nucleus / DNA replication and repair	25	3	5 (3,2)
PF3D7_0512600	Ras-related protein Rab1B	RAB1B	ER-Golgi translocation and quality control	25	5	9 (4,5)
PF3D7_1360900	Polyadenylate-binding protein, putative	PABP	Translation initiation	24	5	11 (5,6)
PF3D7_1353100	Plasmodium exported protein, unknown function	--	Maurer's cleft exported protein	23	3	7 (3,4)
PF3D7_0303000	N-ethylmaleimide-sensitive fusion protein	NSF	Exported vesicle-associated protein	21	4	8 (3,5)
PF3D7_0925900	Conserved *Plasmodium* protein, unknown function	--	No known or predicted function	20	4	8 (4,4)
PF3D7_1135400	Conserved Plasmodium protein, unknown function	--	No known or predicted function	20	2	5 (2,3)
PF3D7_1360800	Falcilysin	FLN	Digestive vacuole, globin peptide degradation	20	4	6 (2,4)
PF3D7_0903200	Ras-related protein Rab7	RAB7	Intracellular traffic, late endosome, digestive vacuole	20	3	5 (3,2)
PF3D7_1212000	Glutathione peroxidase-like thioredoxin peroxidase	TPx(Gl)	Mitochondrial antioxidant system	19	3	7 (3,4)
PF3D7_1343000	Phosphoethanolamine N-methyltransferase	PMT	Methionine and phosphatidylcholine metabolism	19	4	6 (2,4)
PF3D7_0532100	Early transcribed membrane protein 5	ETRAMP5	Parasitophorous vacuole membrane protein	19	3	6 (3,3)
PF3D7_1443900	Heat shock protein 90, putative	HSP90	Chaperone protein, putative apicoplast localization	19	4	7 (4,3)
PF3D7_1416100	Protein SEY1, putative	SEY1	Putative mediator of ER membrane fusion	19	4	8 (4,4)
PF3D7_0532300	*Plasmodium* exported protein (PHISTb), unknown function	--	Exported protein	18	4	6 (2,4)
PF3D7_1431600	Succinyl-CoA ligase [ADP-forming] subunit beta, putative	--	Mitochondrial TCA cycle	17	5	10 (5,5)
PF3D7_1434800	Mitochondrial acidic protein MAM33, putative	--	Mitochondrial matrix, putative	17	6	13 (6,7)
PF3D7_1333000	20 kDa chaperonin	CPN20	Protein chaperone, apicoplast, potentially mitochondrial protein import	15	3	6 (3,3)
PF3D7_0719600	60S ribosomal protein L11a, putative	--	Large ribosomal subunit	15	2	6 (2,4)
PF3D7_0702400	Small exported membrane protein 1	SEMP1	Maurer's cleft exported protein	14	3	7 (3,4)
PF3D7_0831600	Cytoadherence linked asexual protein 8	CLAG8	Putative rhoptry protein	14	4	7 (4,3)
PF3D7_1129900	Major facilitator superfamily-related transporter, putative	MFR5	Putative plasma membrane-localized amino acid transporter	12	4	6 (3,3)

PlasmoDB Gene ID	Gene Name	Abbreviation	Cellular component and/or functional features	Mean fold change	Number of experiments present (of 6)[1]	Number of samples present (of 13 total, 6 WT, 7 mutant)[2]
PF3D7_1143400	Translation initiation factor eIF1A, putative	eIF1A	Translation initiation	11	3	7 (3,4)
PF3D7_1242700	40S ribosomal protein S17, putative	--	Small ribosomal subunit	11	5	11 (5,6)
PF3D7_0807300	Ras-related protein Rab18	RAB18	Intracellular traffic, endocytosis	10	4	9 (4,5)
PF3D7_1126200	40S ribosomal protein S18, putative	--	Small ribosomal subunit	10	4	7 (4,3)
PF3D7_1239700	ATP-dependent zinc metalloprotease FTSH 1	FTSH1	Mitochondrial protein degradation	9	3	7 (3,4)
PF3D7_1365900	Ubiquitin-60S ribosomal protein L40	--	Proteasomal degradation of ubiquitinated proteins	9	3	7 (3,4)
PF3D7_1219100	Clathrin heavy chain, putative	--	Clathrin coat of trans-Golgi network vesicle	9	3	6 (3,3)
PF3D7_1228600	Merozoite surface protein 9	MSP9	Merozoite surface protein	9	4	8 (4,4)
PF3D7_1419200	Thioredoxin-like protein, putative	--	Redox metabolism, putative apicoplast localization	8	2	5 (2,3)
PF3D7_1358800	40S ribosomal protein S15	RPS15	Small ribosomal subunit	8	5	8 (5,3)
PF3D7_0709000	Chloroquine resistance transporter	PfCRT	Digestive vacuole membrane	8	4	9 (4,5)
PF3D7_1346100	Protein transport protein SEC61 subunit alpha	SEC61	ER Sec61 translocon	8	3	7 (3,4)
PF3D7_1441200	60S ribosomal protein L1, putative	--	Large ribosomal subunit	8	5	9 (5,4)
PF3D7_1105400	40S ribosomal protein S4, putative	--	Small ribosomal subunit	8	5	11 (5,6)
PF3D7_1342000	40S ribosomal protein S6	--	Small ribosomal subunit	7	5	8 (3,5)
PF3D7_1211900	Cation ATPase ATP4	ATP4	Sodium-dependent ATPase, plasma membrane	7	4	8 (4,4)
PF3D7_1227100	DNA helicase 60	DH60	Transcription, putative mitochondrial localization	7	3	6 (2,4)
PF3D7_0517300	Serine/arginine-rich splicing factor 1	SR1	Regulation of mRNA splicing, RNA steady state levels	6	3	7 (3,4)
PF3D7_1328300	Conserved *Plasmodium* protein, unknown function	--	No known or predicted function	6	2	5 (2,3)
PF3D7_1036900	Conserved *Plasmodium* protein, unknown function	--	No known or predicted function	5	3	7 (2,5)
PF3D7_1364100	6-cysteine protein	P92	Merozoite surface protein	5	3	5 (2,3)
PF3D7_0102200	Ring-infected erythrocyte surface antigen	RESA	Merozoite dense granules	4	4	8 (4,4)
PF3D7_1404900	Conserved Plasmodium protein, unknown function	--	ER retention sequence, proteome of the parasitophorous vacuole	4	3	5 (2,3)
PF3D7_1127000	Protein phosphatase, putative	--	Digestive vacuole proteome	4	3	6 (3,3)
PF3D7_1344800	Aspartate carbamoyltransferase	ATCase	Ornithine metabolism	4	3	6 (3,3)
PF3D7_1038000	Antigen UB05	--	Digestive vacuole proteome	3	2	5 (2,3)
PF3D7_0601900	Conserved Plasmodium protein, unknown function	--	Maurer's cleft exported protein, upregulated in response to ER stress	3	3	6 (3,3)
PF3D7_1306200	Conserved Plasmodium protein, unknown function	--	Protein released from infected cells during rupture	3	2	5 (2,3)
PF3D7_0731300	*Plasmodium* exported protein (PHISTb), unknown function	G174	Maurer's cleft exported protein	2	4	6 (0,6)

[1]A summary of these experiments can be found in S2 Table.

[2]The 13 samples were derived from Cam3.II^{WT} (4), Cam3.II^{R539T} (4), Cam3.II^{C580Y} (2), CamWT^{WT} (2), and CamWT^{C580Y} (1).
