# Supplementary material for: Insights into the intracellular localization, protein associations and artemisinin resistance properties of Plasmodium falciparum K13
Source: PLoS Pathog. 2020 Apr 20;16(4):e1008482. doi: 10.1371/journal.ppat.1008482 (PMC7192513; doi:10.1371/journal.ppat.1008482)
Supplement: S3 Table — (PDF) [file ppat.1008482.s010.pdf]

S3 Table. PANTHER overrepresentation test for biological processes.

PANTHER Biological Process (GO Category)[1]	Total *P. falciparum* proteins per category	Total proteins in K13 IP list[2]	Expected number of proteins in K13 IP list[3]	Fold enrichment[4]	*p* value[5]	False discovery rate
Ras protein signal transduction (GO:0007265)	13	7	0.40	18	9.8E-07	3.2E-04
Small GTPase mediated signal transduction (GO:0007264)	13	7	0.40	18	9.8E-07	2.4E-04
Tricarboxylic acid cycle (GO:0006099)	9	4	0.27	15	4.3E-04	2.1E-02
Generation of precursor metabolites and energy (GO:0006091)	9	4	0.27	15	4.3E-04	2.0E-02
Nucleoside triphosphate biosynthetic process (GO:0009142)	11	4	0.34	12	7.8E-04	3.2E-02
Nucleotide phosphorylation (GO:0046939)	14	5	0.43	12	1.8E-04	1.9E-02
Phosphorylation (GO:0016310)	14	5	0.43	12	1.8E-04	1.7E-02
Aerobic respiration (GO:0009060)	12	4	0.37	11	1.0E-03	4.0E-02
Pyruvate metabolic process (GO:0006090)	12	4	0.37	11	1.0E-03	3.8E-02
Nucleotide catabolic process (GO:0009166)	12	4	0.37	11	1.0E-03	3.7E-02
Carboxylic acid metabolic process (GO:0019752)	16	5	0.49	10	3.0E-04	2.1E-02
Coenzyme metabolic process (GO:0006732)	17	5	0.52	9.6	3.8E-04	2.1E-02
Organic acid metabolic process (GO:0006082)	17	5	0.52	9.6	3.8E-04	2.0E-02
Oxoacid metabolic process (GO:0043436)	17	5	0.52	9.6	3.8E-04	1.9E-02
Nucleoside triphosphate metabolic process (GO:0009141)	14	4	0.43	9.4	1.6E-03	4.6E-02
Cellular respiration (GO:0045333)	24	6	0.73	8.2	2.0E-04	1.8E-02
Energy derivation by oxidation of organic compounds (GO:0015980)	24	6	0.73	8.2	2.0E-04	1.6E-02
Phosphate-containing compound metabolic process (GO:0006796)	26	6	0.79	7.6	2.9E-04	2.2E-02
Oxidation-reduction process (GO:0055114)	27	6	0.82	7.3	3.5E-04	2.3E-02
Carbohydrate metabolic process (GO:0005975)	27	6	0.82	7.3	3.5E-04	2.1E-02
Coenzyme biosynthetic process (GO:0009108)	24	5	0.73	6.8	1.4E-03	4.2E-02
Response to chemical (GO:0042221)	25	5	0.76	6.6	1.7E-03	4.5E-02
Phosphorus metabolic process (GO:0006793)	31	6	1.0	6.3	6.7E-04	2.8E-02
Cofactor metabolic process (GO:0051186)	59	11	1.8	6.1	5.2E-06	1.0E-03
Response to stimulus (GO:0050896)	49	9	1.5	6.0	4.3E-05	5.2E-03
Nucleobase-containing small molecule metabolic process (GO:0055086)	50	7	1.5	4.6	1.3E-03	4.1E-02
Small molecule metabolic process (GO:0044281)	58	8	1.8	4.5	6.6E-04	2.9E-02
Cellular localization (GO:0051641)	195	15	6.0	2.5	1.3E-03	4.0E-02
Cellular metabolic process (GO:0044237)	339	26	10	2.5	3.3E-05	4.5E-03
Biological regulation (GO:0065007)	215	16	6.6	2.4	1.2E-03	4.1E-02
Cellular component organization (GO:0016043)	247	18	7.5	2.4	1.1E-03	3.7E-02
Cellular process (GO:0009987)	833	57	25	2.2	3.6E-09	1.7E-06
Localization (GO:0051179)	346	22	11	2.1	1.3E-03	4.1E-02
Metabolic process (GO:0008152)	904	50	28	1.8	2.2E-05	3.5E-03
Organic substance metabolic process (GO:0071704)	773	39	24	1.7	1.6E-03	4.6E-02

[1]Refers to GO-Slim Biology Process database from PANTHER version 14.1 (http://pantherdb.org). Released 2019-03-12.

[2]Immunoprecipitated proteins are listed in Table 1 or S3 Table. Of the 173 listed in those tables, 166 were assigned to GO categories by PANTHER.

[3]This calculation was based on the PANTHER estimation of 5,500 *P. falciparum* proteins. This is similar to the lower estimate of 4,800 proteins in the Malaria Parasite Metabolic Pathways database (http://mpmp.huji.ac.il).

[4]Enrichment was calculated as the number of proteins identified in the K13 IP list divided by the number expected if the IP output were random.

[5]Overrepresentation was determined by Fisher's exact tests with false discovery rate controls.

GO, gene ontology; IP, immunoprecipitation.
