# Supplementary material for: Insights into the intracellular localization, protein associations and artemisinin resistance properties of Plasmodium falciparum K13
Source: PLoS Pathog. 2020 Apr 20;16(4):e1008482. doi: 10.1371/journal.ppat.1008482 (PMC7192513; doi:10.1371/journal.ppat.1008482)
Supplement: S4 Table — (PDF) [file ppat.1008482.s011.pdf]

S4 Table. PANTHER overrepresentation test for cellular components.

PANTHER Cellular Component (GO Category)[1]	Total *P. falciparum* proteins per category	Total proteins in K13 IP list[2]	Expected number of proteins in K13 IP list[3]	Fold enrichment[4]	*p* value[5]	False discovery rate
Oxidoreductase complex (GO:1990204)	5	4	0.15	26	8.3E-05	2.1E-03
Apical part of cell (GO:0045177)	5	3	0.15	20	1.3E-03	2.6E-02
Mitochondrial protein complex (GO:0098798)	9	4	0.27	15	4.3E-04	1.0E-02
Cytosolic small ribosomal subunit (GO:0022627)	35	10	1.1	9.4	5.1E-07	1.6E-05
Cytosolic ribosome (GO:0022626)	82	14	2.5	5.6	6.7E-07	1.9E-05
Cytosolic part (GO:0044445)	98	16	3.0	5.4	1.8E-07	6.0E-06
Cytosol (GO:0005829)	250	34	7.6	4.5	9.2E-13	3.6E-11
Mitochondrion (GO:0005739)	77	9	2.4	3.8	9.2E-04	2.0E-02
Cytoplasmic part (GO:0044444)	579	56	18	3.2	5.7E-15	2.9E-13
Cell (GO:0005623)	1008	87	31	2.8	7.3E-22	2.2E-19
Cell part (GO:0044464)	1004	86	31	2.8	2.5E-21	3.9E-19
Intracellular (GO:0005622)	940	79	29	2.8	1.2E-18	1.2E-16
Cytoplasm (GO:0005737)	859	72	26	2.8	1.4E-16	8.3E-15
Intracellular part (GO:0044424)	905	75	28	2.7	3.7E-17	2.8E-15

[1]Refers to GO-Slim Cellular Component database from PANTHER version 14.1 (http://pantherdb.org). Released 2019-03-12.

[2]Immunoprecipitated proteins are listed in Table 1 or S3 Table. Of the 173 listed in those tables, 166 were assigned to GO categories by PANTHER.

[3]This calculation was based on the PANTHER estimation of 5,500 *P. falciparum* proteins. This is similar to the lower estimate of 4,800 proteins in the Malaria Parasite Metabolic Pathways database (http://mpmp.huji.ac.il).

[4]Enrichment was calculated as the number of proteins identified in the K13 IP list divided by the number expected if the IP output were random.

[5]Overrepresentation was determined by Fisher's exact tests with false discovery rate controls.

GO, gene ontology; IP, immunoprecipitation.
