# Supplementary material for: Insights into the intracellular localization, protein associations and artemisinin resistance properties of Plasmodium falciparum K13
Source: PLoS Pathog. 2020 Apr 20;16(4):e1008482. doi: 10.1371/journal.ppat.1008482 (PMC7192513; doi:10.1371/journal.ppat.1008482)
Supplement: S5 Table — (PDF) [file ppat.1008482.s012.pdf]

S5 Table. Pearson correlation coefficient values for IFA studies.

			0h post treatment		3h post treatment		12h post treatment		24h post treatment			1h post treatment	
Parasite line[1]	Antibodies used for staining	Treatment[2]	N[3]	PCC median [IQR]	N	PCC median [IQR]	N	PCC median [IQR]	N	PCC median [IQR]	Treatment[4]	N	PCC median [IQR]
Cam3.II^{R539T}	α-K13 (E3) / α-Rab5A	DMSO	20	0.47 [0.38-0.67]	--	--	20	0.37 [0.21-0.52]	--	--	EtOH	--	--
		DHA (700 nM)	21	0.44 [0.33-0.58]	--	--	22	0.43 [0.28-0.65]	--	--	BFA (5 µg/ml)	--	--
Cam3.II^{WT}	α-K13 (E3) / α-Rab5A	DMSO	23	0.47 [0.39-0.57]	--	--	23	0.53 [0.46-0.61]	--	--	EtOH	--	--
		DHA (700 nM)	21	0.50 [0.37-0.58]	--	--	37	0.65 [0.53-0.71]	--	--	BFA (5 µg/ml)	--	--
Cam3.II^{R539T}	α-K13 (E3) / α-Rab5B	DMSO	16	0.34 [0.30-0.57]	--	--	--	--	--	--	EtOH	--	--
		DHA (700 nM)	14	0.34 [0.20-0.42]	--	--	--	--	--	--	BFA (5 µg/ml)	--	--
Cam3.II^{WT}	α-K13 (E3) / α-Rab5B	DMSO	21	0.41 [0.30-0.49]	--	--	--	--	--	--	EtOH	--	--
		DHA (700 nM)	17	0.43 [0.27-0.55]	--	--	--	--	--	--	BFA (5 µg/ml)	--	--
Cam3.II^{R539T}	α-K13 (E3) / α-Rab5C	DMSO	11	0.33 [0.25-0.43]							EtOH	--	--
		DHA (700 nM)	11	0.27 [0.19-0.47]	--	--	--	--	--	--	BFA (5 µg/ml)	--	--
Cam3.II^{WT}	α-K13 (E3) / α-Rab5C	DMSO	14	0.41 [0.34-0.57]	--	--	--	--	--	--	EtOH	--	--
		DHA (700 nM)	15	0.48 [0.37-0.57]	--	--	--	--	--	--	BFA (5 µg/ml)	--	--
Cam3.II^{R539T}	α-K13 (E3) / α-Rab7	DMSO	11	0.67 [0.56-0.74]	--	--	--	--	--	--	EtOH	--	--
		DHA (700 nM)	13	0.59 [0.49-0.70]	--	--	--	--	--	--	BFA (5 µg/ml)	--	--
Cam3.II^{WT}	α-K13 (E3) / α-Rab7	DMSO	13	0.68 [0.58-0.73]	--	--	--	--	--	--	EtOH	--	--
		DHA (700 nM)	13	0.68 [0.56-0.75]	--	--	--	--	--	--	BFA (5 µg/ml)	--	--
Dd2^{R539T} Rab6-GFP	α-K13 (E3) / α-GFP	DMSO	21	0.52 [0.46-0.58]	--	--	--	--	--	--	EtOH	--	--
		DHA (700 nM)	28	0.64 [0.57-0.76]	--	--	--	--	--	--	BFA (5 µg/ml)	--	--
Dd2^{WT} Rab6-GFP	α-K13 (E3) / α-GFP	DMSO	11	0.53 [0.37-0.64]	--	--	--	--	--	--	EtOH	--	--
		DHA (700 nM)	10	0.73 [0.61-0.83]	--	--	--	--	--	--	BFA (5 µg/ml)	--	--
Cam3.II^{R539T}	α-K13 (E3) / α-Rab11A	DMSO	18	0.83 [0.60-0.95]	--	--	12	0.72 [0.56-0.78]	--	--	EtOH	--	--
		DHA (700 nM)	13	0.84 [0.76-0.92]	--	--	18	0.70 [0.63-0.85]	--	--	BFA (5 µg/ml)	--	--
Cam3.II^{WT}	α-K13 (E3) / α-Rab11A	DMSO	14	0.60 [0.44-0.74]	--	--	17	0.61 [0.51-0.66]	--	--	EtOH	--	--
		DHA (700 nM)	15	0.86 [0.75-0.91]	--	--	14	0.64 [0.55-0.70]	--	--	BFA (5 µg/ml)	--	--
Dd2^{WT} Sec24A-GFP	α-K13 (E3) / α-GFP	DMSO	11	0.67 [0.42-0.72]	--	--	--	--	--	--	EtOH	--	--
		DHA (700 nM)	12	0.60 [0.49-0.68]	--	--	--	--	--	--	BFA (5 µg/ml)	--	--
Cam3.II^{R539T}	α-K13 (E3) / α-BiP	DMSO	19	0.72 [0.55-0.85]	8	0.59 [0.51-0.80]	11	0.78 [0.71-0.93]	10	0.47 [0.28-0.54]	EtOH	20	0.64 [0.55-0.67]
		DHA (700 nM)	15	0.65 [0.49-0.69]	9	0.60 [0.52-0.66]	10	0.82 [0.76-0.89]	11	0.75 [0.57-0.76]	BFA (5 µg/ml)	23	0.85 [0.68-0.92]
Cam3.II^{WT}	α-K13 (E3) / α-BiP	DMSO	20	0.64 [0.48-0.74]	9	0.61 [0.48-0.65]	11	0.77 [0.65-0.80]	9	0.35 [0.13-0.43]	EtOH	17	0.68 [0.54-0.73]
		DHA (700 nM)	19	0.62 [0.49-0.70]	10	0.65 [0.55-0.75]	10	0.58 [0.41-0.73]	13	0.45 [0.33-0.52]	BFA (5 µg/ml)	17	0.56 [0.29-0.70]
Cam3.II^{R539T}	α-K13 (E3) / α-ERD2	DMSO	9	0.39 [0.29-0.52]	--	--	--	--	--	--	EtOH	20	0.42 [0.31-0.57]
		DHA (700 nM)	10	0.40 [0.21-0.53]	--	--	--	--	--	--	BFA (5 µg/ml)	23	0.47 [0.33-0.58]
Cam3.II^{WT}	α-K13 (E3) / α-ERD2	DMSO	9	0.44 [0.31-0.57]	--	--	--	--	--	--	EtOH	26	0.39 [0.29-0.52]
		DHA (700 nM)	9	0.41 [0.24-0.43]	--	--	--	--	--	--	BFA (5 µg/ml)	23	0.40 [0.35-0.55]
Cam3.II^{R539T}	α-K13 (E3) / α-TRiC	DMSO	22	0.62 [0.50-0.69]	--	--	--	--	--	--	EtOH	--	--
		DHA (700 nM)	19	0.63 [0.50-0.75]	--	--	--	--	--	--	BFA (5 µg/ml)	--	--
Cam3.II^{WT}	α-K13 (E3) / α-TRiC	DMSO	23	0.61 [0.51-0.71]	--	--	--	--	--	--	EtOH	--	--
		DHA (700 nM)	21	0.68 [0.53-0.77]	--	--	--	--	--	--	BFA (5 µg/ml)	--	--

[1]Cam3.II^{WT} was previously referred to as Cam3.II^{rev} (clone C2; Straimer *et al.*, 2015; PMID 25502314).

[2]Very early ring-stage parasites (0-3 hpi) were exposed to 700 nM DHA or DMSO vehicle control for 6h, after which compound was removed by washout.

[3]N, number of individual parasites used for quantitative imaging-based analyses. Individual parasites were analyzed from one to two independent experiments.

[4]Very early ring-stage parasites (0-3 hpi) were exposed to BFA at 5 µg/ml or EtOH vehicle control for 6h, after which compound was removed by washout.

BFA, Brefeldin A; DHA, dihydroartemisinin; DMSO, dimethyl sulfoxide; EtOH, ethanol; IQR, interquartile range; PCC, Pearson correlation coefficient.
