# Supplementary material for: Insights into the intracellular localization, protein associations and artemisinin resistance properties of Plasmodium falciparum K13
Source: PLoS Pathog. 2020 Apr 20;16(4):e1008482. doi: 10.1371/journal.ppat.1008482 (PMC7192513; doi:10.1371/journal.ppat.1008482)
Supplement: S6 Table — (PDF) [file ppat.1008482.s013.pdf]

S6 Table. Pearson correlation coefficient values for MitoTracker Deep Red imaging studies.

				0h post treatment		12h post treatment	
**Parasite line**	**Antibody/Dye used for staining**	**Treatment[1]**	**Duration of Treatment**	**N[2]**	**PCC median [IQR]**	**N**	**PCC median [IQR]**
$NF54^{WT}attB\text{-}GFP\text{-}K13^{WT}$	α-GFP / MitoTracker Deep Red	DMSO	6h	31	0.25 [0.10-0.41]	18	0.44 [0.16-0.60]
		DHA (700 nM)	6h	33	0.55 [0.33-0.76]	20	0.84 [0.74-0.92]
$NF54^{WT}attB\text{-}3HA\text{-}K13^{C580Y}$	α-HA / MitoTracker Deep Red	DMSO	6h	16	0.34 [0.22-0.53]	--	--
		DHA (700 nM)	6h	19	0.56 [0.42-0.61]	--	--
$Cam3.II^{R539T}$	α-K13 (E3) / MitoTracker Deep Red	DMSO	6h	20	-0.03 [-0.11-0.13]	19	0.031 [-0.04-0.10]
		DHA (700 nM)	6h	23	0.22 [0.09-0.40]	20	0.37 [0.14-0.52]
$Cam3.II^{WT}$	α-K13 (E3) / MitoTracker Deep Red	DMSO	6h	18	0.11 [0.02-0.24]	21	0.21 [0.17-0.33]
		DHA (700 nM)	6h	22	0.24 [0.07-0.39]	22	0.42 [0.36-0.53]
$Cam3.II^{R539T}$	α−K13 (E3) / MitoTracker Deep Red	DMSO	4h	11	0.10 [-0.05-0.17]	--	--
		DHA (60 nM)	4h	10	0.05 [-0.15-0.13]	--	--
		ATQ (100 nM)	4h	13	0.06 [-0.04-0.20]	--	--
		ATQ (1200 nM)	4h	12	0.03 [-0.06-0.17]	--	--
		DHA 60 nM + ATQ 100 nM	4h	13	0.33 [0.14-0.41]	--	--
		DHA 60 nM + ATQ 1200 nM	4h	12	0.30 [0.12-0.57]	--	--
$Cam3.II^{WT}$	α-K13 (E3) / MitoTracker Deep Red	DMSO	4h	11	0.25 [-0.01-0.35]	--	--
		DHA (60 nM)	4h	10	0.08 [0.00-0.37]	--	--
		ATQ (100 nM)	4h	12	0.28 [0.06-0.35]	--	--
		ATQ (1200 nM)	4h	12	0.27 [0.09-0.31]	--	--
		DHA 60 nM + ATQ 100 nM	4h	12	0.10 [0.02-0.23]	--	--
		DHA 60 nM + ATQ 1200 nM	4h	9	0.38 [0.23-0.41]	--	--
$Cam3.II^{R539T}$	α-ERD2 / MitoTracker Deep Red	DMSO	6h	13	0.38 [0.18-0.68]	--	--
		DHA (700 nM)	6h	12	0.43 [0.27-0.52]	--	--
$Cam3.II^{WT}$	α-ERD2 / MitoTracker Deep Red	DMSO	6h	15	0.51 [0.25-0.67]	--	--
		DHA (700 nM)	6h	13	0.56 [0.33-0.71]	--	--
$Cam3.II^{R539T}$	α-TRiC / MitoTracker Deep Red	DMSO	6h	13	0.44 [0.32-0.62]	--	--
		DHA (700 nM)	6h	14	0.38 [0.26-0.56]	--	--
$Cam3.II^{WT}$	α-TRiC / MitoTracker Deep Red	DMSO	6h	13	0.55 [0.30-0.68]	--	--
		DHA (700 nM)	6h	16	0.51 [0.25-0.67]	--	--
$Cam3.II^{R539T}$	Rab5A / MitoTracker Deep Red	DMSO	6h	12	0.46 [0.19-0.57]	--	--
		DHA (700 nM)	6h	13	0.35 [0.30-0.55]	--	--
$Cam3.II^{WT}$	Rab5A / MitoTracker Deep Red	DMSO	6h	12	0.37 [0.23-0.45]	--	--
		DHA (700 nM)	6h	14	0.44 [0.21-0.56]	--	--
$Cam3.II^{R539T}$	Rab11A / MitoTracker Deep Red	DMSO	6h	14	0.29 [0.17-0.70]	--	--
		DHA (700 nM)	6h	15	0.45 [0.18-0.71]	--	--
$Cam3.II^{WT}$	Rab11A / MitoTracker Deep Red	DMSO	6h	13	0.10 [0.00-0.57]	--	--
		DHA (700 nM)	6h	11	0.35 [0.06-0.43]	--	--

[1]Very early ring-stage parasites (0-3 hpi) were pulsed with indicated compound(s) for 4 or 6h, after which compound(s) was/were removed by washout.

[2]N, number of individual parasites used for analyses. Individual parasites were analyzed from one to two independent experiments.

ATQ, atovaquone; DHA, dihydroartemisinin; DMSO, dimethyl sulfoxide; IQR, interquartile range; PCC, Pearson correlation coefficient.
