# Supplementary material for: Insights into the intracellular localization, protein associations and artemisinin resistance properties of Plasmodium falciparum K13
Source: PLoS Pathog. 2020 Apr 20;16(4):e1008482. doi: 10.1371/journal.ppat.1008482 (PMC7192513; doi:10.1371/journal.ppat.1008482)
Supplement: S7 Table — (PDF) [file ppat.1008482.s014.pdf]

S7 Table. Oligonucleotides used in this study.

Name[1]	Nucleotide sequence (5'-3')	Description	Lab name
--	AGATCTATGGAAGGAGAAAAAGTAAAAACAAAAGC	*K13* fwd	p3947
--	CTCGAGTTATATATTTGCTATTAAAACGGAGTGACC	*K13* rev	p3948
--	CTGGGCCCATACCATGTTGAATTGGTGACAGG	*K13* 5'UTR fwd	p4376
--	CCTAGGAATTATAATTTAATTAACAAAAACATAATAAATGAATG	*K13* 5'UTR rev	p4377
--	CTCAGATCTGGTGGAATGGATGAATTTCAAAGTAAGC	*Rab6* fwd	poML214
--	CCCTCGAGTTAACATAAACATTTACTTAACATATTTTTGTC	*Rab6* rev	poML204
--	CCGGGCCCTCATGCCCTAATAAAAAGTTGCC	*Sec12* 5'UTR fwd	p1144
--	CGCCTAGGTTTGTTCTTTTTATAAATAACA	*Sec12* 5'UTR rev	p1263
p1	CCATGTTGAATTGGTGACAGG	*K13* 5'UTR fwd	p7370
p2	CTAAGAATATTCTTCCTTGTTTATCTCTGG	*K13* rev	p6456
p3	GATGCAAATATTGCTACTGAAACTATGATTG	*K13* fwd	p6457
p4	ATTACCGATATCTGATGTATCATAGGTTAAAGCGTCA	*K13* 3'UTR rev	p6709
p5	GAGTTTGTAACAGCTGCTGGG	*GFP* fwd	p7363
p6	CGACTAGTTAATAAAGGGCAC	*EF1α* fwd	p6455
p7	GTATATTGGGGTGATGATAAAATGAAAG	*hsp86* 3'UTR rev	p6458
p8	GAAAATATTATTACAAAGGGTGAGG	*cg6* fwd	p4092
p9	TTAGCTAATTCGCTTGTAAGA	*bsd* rev	p4093
p10	CGGTCACAGCTTGTCTGTAAGCGG	pDC2 backbone fwd	p5863
p11	CTCTTCTACTCTTTCGAATTC	*cg6* rev	p4094

[1]Primer name refers to numbered primers depicted in S1 Fig.

fwd, forward; rev, reverse; UTR, untranslated region.
